# Synthetically Versatile Nitrogen Acyclic Carbene Stabilized Gold Nanoparticles

**DOI:** 10.1002/chem.202003679

**Published:** 2020-11-18

**Authors:** Guilherme M. D. M. Rúbio, Bernhard K. Keppler, Jia Min Chin, Michael R. Reithofer

**Affiliations:** ^1^ Institute of Inorganic Chemistry Faculty of Chemistry University of Vienna Waehringer Strasse 42 1090 Vienna Austria; ^2^ Institute of Physical Chemistry Faculty of Chemistry University of Vienna Waehringer Strasse 42 1090 Vienna Austria

**Keywords:** gold nanoparticles, *N*-acyclic carbenes, *N*-heterocyclic carbenes, nonsymmetric carbenes, water dispersible nanoparticles

## Abstract

*N*‐heterocyclic carbenes (NHCs) have received significant attention as gold nanoparticle stabilizers due to their strong binding affinity towards gold. However, their tunability is limited by the difficulty in obtaining nonsymmetric NHCs. In this regard, *N*‐acyclic carbenes (NACs) are attractive alternatives due to their high synthetic versatility, allowing easy tuning of their steric and electronic properties towards specific applications. This work reports the first series of stable and monodisperse NAC‐functionalized gold nanoparticles. These particles with sizes ranging 3.8 to 11.6 nm were characterized using NMR, UV/Vis and TEM. The nanoparticles display good stability at elevated temperatures and for extended periods both dried or dispersed in a medium, as well as in the presence of exogenous thiols. Importantly, these NAC‐stabilized gold nanoparticles offer a promising and versatile alternative to NHC‐stabilized gold nanoparticles.

## Graphical abstract

**Gold**: *N*‐acyclic carbenes (NACs) have been shown to function as versatile ligand alternatives to *N*‐heterocyclic carbenes in the synthesis of a new series of stable plasmonic gold nanoparticles. The ease of preparing nonsymmetric NAC ligands and their synthetic tunability renders them especially attractive as gold nanoparticle stabilizers and opens up new possibilities in the design of gold nanoparticles for user‐specified functions (see figure).

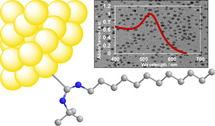

Gold nanoparticles are amongst the most well‐studied metal‐based nanostructures, which find applications in sensing,^[1]^ catalysis,^[2]^ drug delivery,^[3]^ bioimaging,^[4]^ and photonics.^[5]^ Although considerable efforts were made to understand, and tailor, the shape and properties of gold nanoparticles, their surface chemistry remained almost unchanged for decades.^[6]^ Only recent interest in *N*‐heterocyclic carbenes as an alternative to thiol based surfactants has opened novel possibilities in this area of nanochemistry.^[7]^ Although NHC‐stabilized AuNPs (NHCAuNPs) have shown great promise for the development of novel nanomaterials, NHCAuNPs face several drawbacks, which need to be addressed before they can be more widely adopted and applied.[^7d^, ^8^] While NHCAuNPs have shown excellent stability in a variety of environmental conditions, ranging from high temperature to dispersibility in high ionic strength solutions, most of the known NHCAuNP examples readily degrade in the presence of thiols. To obtain highly stable NHCAuNPs with improved thiol stability therefore necessitates careful design of the NHC ligand. However, synthetic access to functionalized or nonsymmetric NHCs can be difficult, thereby limiting the versatility of NHCAuNPs. Developing novel nanomaterials which enjoy the stability afforded by carbene ligands whilst possessing flexibility with regards to ligand design and functionalization would therefore significantly broaden the possible applications of carbene stabilized AuNPs.

Nitrogen acyclic carbene (NAC) gold complexes are well known and have especially been explored for catalytic applications.^[9]^ As NAC gold complexes are commonly synthesized through simple addition of an amine to an electrophilic isocyanide gold(I) complex, the wide variety of amines to choose from lends versatility to the design and synthesis of NAC gold complexes, allowing researchers to tailor the ligands to specific functions. Further, this synthetic approach also means that nonsymmetric NACs are easily prepared. Surprisingly, however, NAC‐stabilized AuNPs have not been reported thus far. We report here for the first time the use of NACs as surfactant ligands to stabilize plasmonic AuNPs which display good stability towards exogenous thiols. The methodology developed here should allow rapid access to nonsymmetric structures, thus opening new possibilities in gold nanomaterial development.

The NAC gold(I) chloride compounds (**3 a**–**3 e**) were synthesized from isocyanide gold(I) chloride precursors, following established reaction procedures (Scheme 1).^[10]^ It is worth noting that one of the characteristics of NAC gold(I) chloride complexes is the presence of conformational isomers or rotamers.^[11]^ This is especially evident in the ^1^H‐NMR spectrum of compound **3 a** and **3 b** (Figure S5 and S7, Supporting Information). Generally, two rotamers are observed in the ^1^H‐NMR (*syn*‐*anti* and *anti*–*syn*), depending if the ^*t*^butyl group is *anti* or *syn* to the gold. Both NAC residues R_1_ and R_2_ cannot be in an *anti*‐position to the gold, due to steric reasons. As a result, two sets of NMR signals can be detected. For the residue R_1_, ^*t*^butyl and cyclohexyl groups were chosen to allow comparison of gold nanoparticles bearing ligands with different steric requirements. For the R_2_ groups, lipophilic amines bearing dodecyl or octyl hydrocarbon chains were used for the ligand synthesis to study the impact of stabilizers bearing different hydrocarbon chain lengths on the gold nanoparticle stability. Diethylamine was also utilized to prepare the compound **3 c** to demonstrate the flexibility regarding the choice of primary and secondary amine reagents for NAC ligand synthesis.

**Scheme 1:**
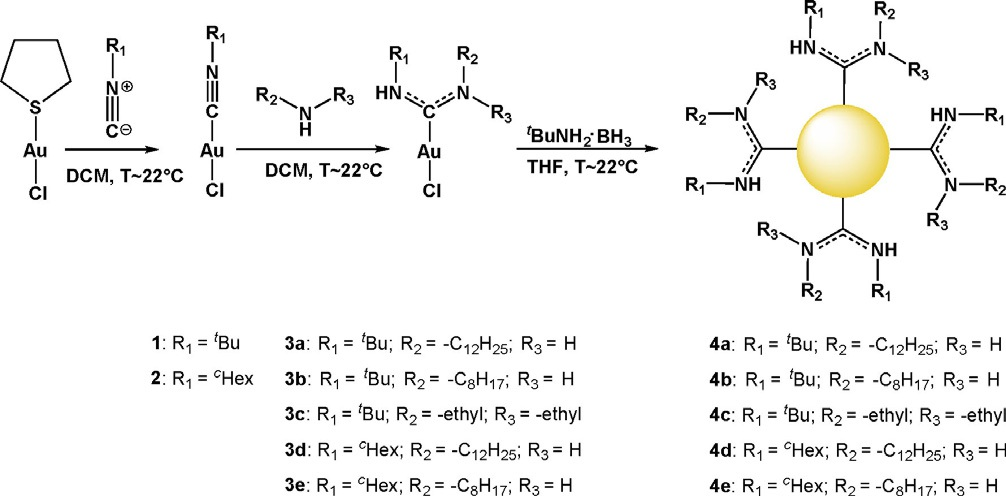
General procedure for the synthesis of NAC‐stabilized gold nanoparticles.

NAC gold(I) compounds were reduced using *t*BuNH_2_⋅BH_3_, whereby upon addition of a solution of *t*BuNH_2_⋅BH_3_ to a stirred solution of compound **3 a**–**e** in THF, a deep red color is observed within minutes, indicating the formation of NACAuNPs. The red color was maintained as the reaction was stirred for several hours and no indication of precipitation was found. In order to obtain pure NACAuNPs, the reaction mixture was quenched with a few drops of water, then the mixture was concentrated under reduced pressure and finally cleaned through several washing and centrifugation steps using ethyl acetate as solvent. Interestingly, the reduction of **3 c** yielded nanoparticles which are non‐dispersible in ethyl acetate. Thus, **4 c** was purified via centrifugation from THF to remove the soluble side products, and stable nanoparticles were obtained after freeze drying from water.

The successful formation of AuNPs was indicated by the presence of a plasmon resonance band (PRB) in the UV/Vis spectrum at 517 nm (**4 a**), 522 nm (**4 b**), 523 nm (**4 c**) 520 nm (**4 d**) and 525 nm (**4 e**) (see Figure 1 for compound **4 a** and Figure S25, S27, S29 and S31 for **4 b**, **4 c**, **4 d** and **4 e**, respectively). To show the binding of the NAC ligands onto the AuNP surface, ^1^H and ^13^C NMR spectroscopy was carried out. Hereby ^13^C NMR is an especially useful method for providing insight into transformations of the chemical species present, with a low field shift of the carbene carbon signal indicating a change in its chemical environment, thus suggesting the successful formation of NACAuNPs.^[8h]^ The ^13^C carbene NAC signal for complex **3 b** (Figure 2), **3 c** and **3 e** was detected as a weak signal at 191.0, 189.5 and 190.7 ppm, respectively. After reduction a low field shift to 206.3 (**4 b**) (Figure 2) and 206.0 ppm (**4 e**) respectively was detected, whereas **4 c** displayed a less pronounced low field shift at 200.3 ppm, indicating NACAuNP formation. Similar shifts between the molecular and the nanoparticle species have been reported for NHC‐based particles, although the effect is less pronounced than in our case.[^7d^, ^8h^] Despite the absence of a detectable carbene ^13^C signal for compounds **3 a** and **3 d**, a chemical shift of the carbene NAC was detected at 205.9 ppm for nanoparticle **4 d**, which is in good agreement with the other signals observed. Nonetheless, the other shifts observed by ^1^H and ^13^C NMR still support the presence of the NAC ligand in **3 a** and **3 d**, even though the carbene signal could not be directly observed. The nanoparticles **4 a**, **4 b**, **4 d** and **4 e** were dispersible in toluene but not in water. Interestingly, although **4 c** was readily dispersed in water, it was not dispersible in most organic solvents. This is attributed to the lack of long hydrocarbon chains in **4 c**, compared to the other nanoparticles. Further, zeta potential measurements indicated that **4 c** bears a negative zeta potential of −17.1 mV which also contributes to its water dispersibility.

**Figure 1:**
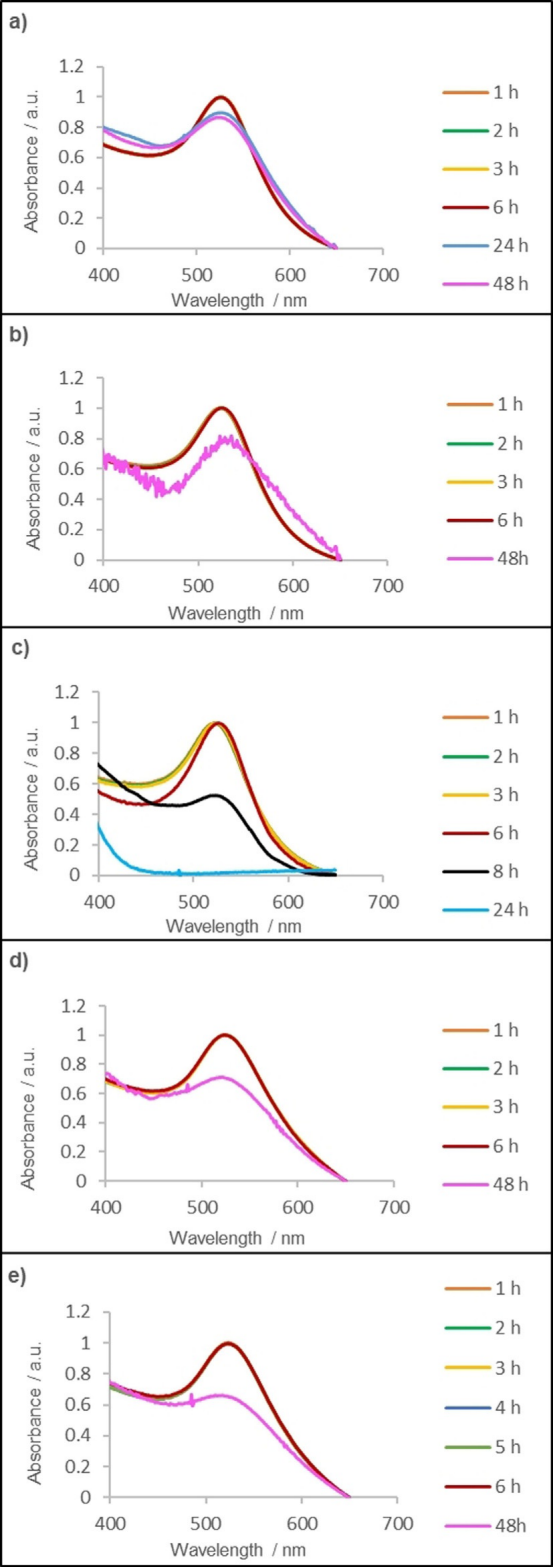
UV/Vis spectra of **4 a** in toluene at a) 25 °C b) 50 °C c) 80 °C and UV/Vis spectra of **4 a** in toluene in the presence of a 10 mm 1‐dodecanethiol at d) 25 °C and e) 50 °C.

**Figure 2:**
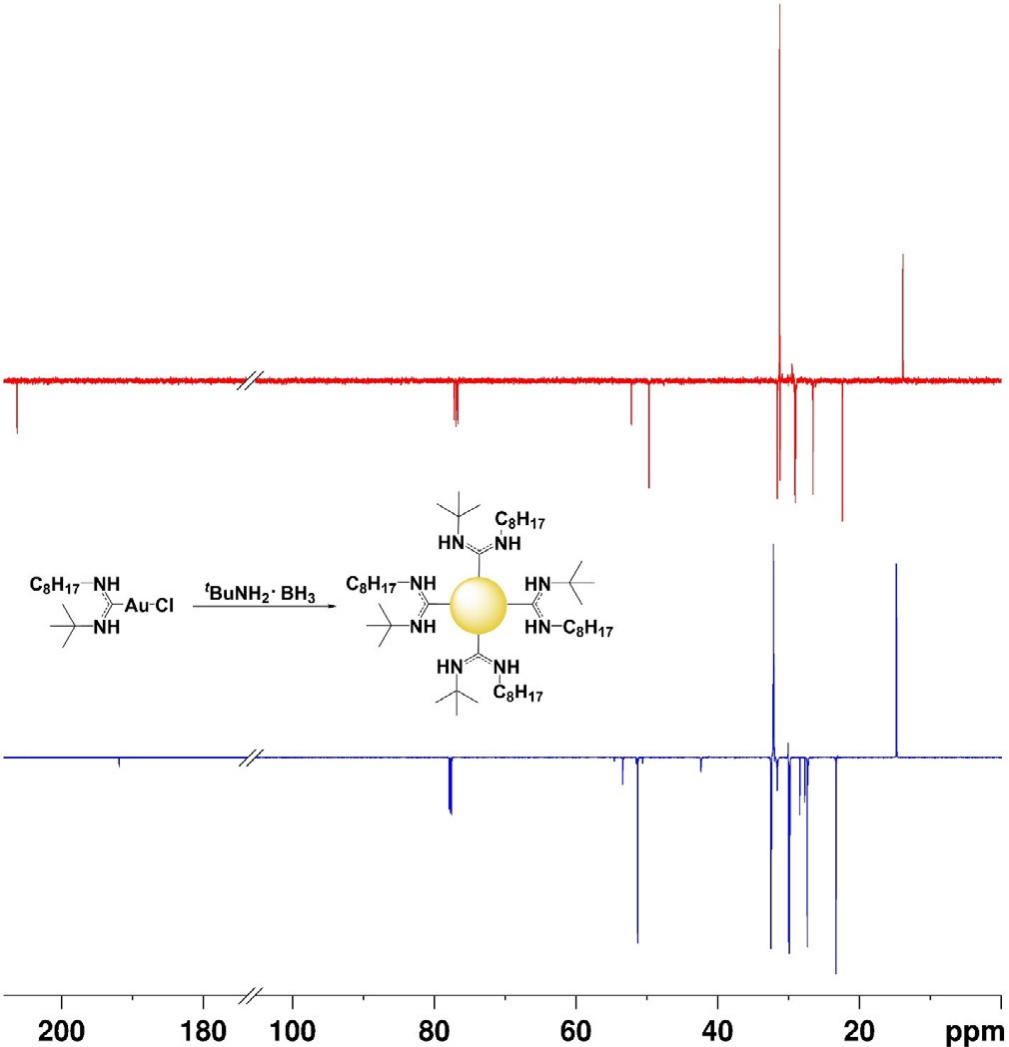
^13^C NMR spectrum of compound **3 b** (blue) and **4 b** (red).

Further, thermogravimetric analysis was performed to estimate the amount of NAC ligand present on the AuNPs surface. TGA analysis showed that the particles contained 55.9 % (**4 a**), 47.8 % (**4 b**), 56.4 % (**4 c**), 52.1 % (**4 d**) and 47.1 % (**4 e**) organic component by weight, respectively (Figures S33–S37).

TEM images (Figure 3) from films obtained via drop casting of solutions of NPs **4 a**, **4 b**, **4 d** and **4 e**, in toluene, and **4 c** in water, and drying showed well defined spherical NPs for all samples. Images of samples **4 a**, **4 b**, **4 c** and **4 e** showed narrowly dispersed AuNPs with a size range of 3.9±0.7 nm (**4 a**), 3.8±0.7 nm (**4 b**), 4.5±0.8 nm (**4 c**) and 3.8±0.7 nm (**4 e**) respectively, whereas the reduction of complex **3 d** resulted in AuNPs which are significantly larger (11.6±3.2 nm). Further, sample **4 d** also showed a significantly broader size distribution of ±28 % when compared to **4 a**, **4 b**, **4 c** and **4 e**, respectively. It is worth noting, when comparing the TEM images **4 a**, **4 b**, **4 d** and **4 e** that cyclohexyl bearing NPs (**4 d** and **4 e**) are generally less regular in shape. This is especially obvious with sample **4 d**, where triangles and rods are present. Based on this observation, it appears that the substituents R_1_, R_2_ and R_3_ play a crucial role in determining the shape of the resulting NACAuNPs. When the R_1_ substituent is cyclohexyl, instead of the more sterically demanding ^*t*^butyl group, less regularly shaped NPs are obtained.

**Figure 3:**
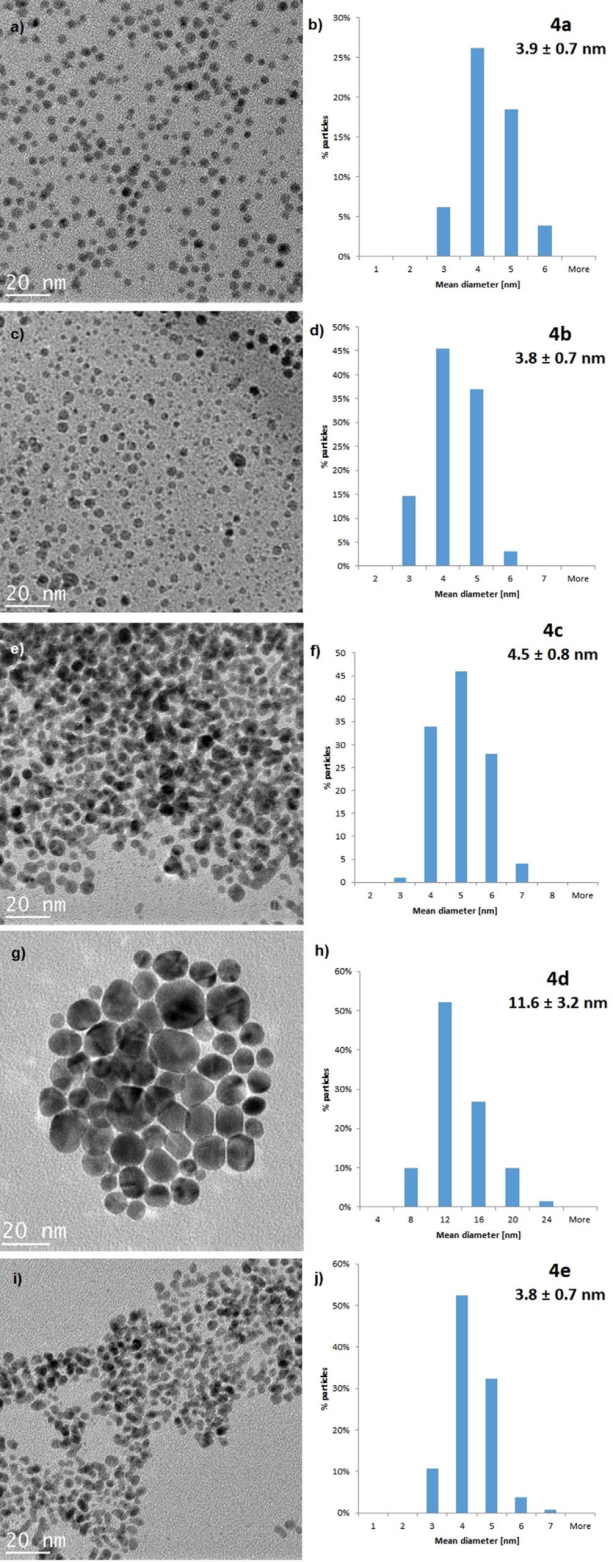
TEM images of NACAuNPs and their size distribution; **4 a** (a and b), **4 b** (c and d), **4 c** (e and f), **4 d** (g and h) and **4 e** (i and j).

To investigate thermal and chemical stability of **4 a**, **4 b**, **4 d** and **4 e**, NACAuNPs were either dispersed in toluene or in a 10 mm solution of 1‐dodecanethiol in toluene. As **4 c** is water dispersible, stability tests were performed in water or in a 10 mm solution of glutathione (GSH) in water at pH 7.4 instead. For thermal stability testing, the resulting solutions were heated at 25, 50 and 80 °C up to 48 h or until degraded, whereas samples containing 1‐dodecathiol were studied at 25 and 50 °C up to 48 h. The stability of all samples was monitored through any changes of the RPB by UV/Vis spectroscopy, whereby a red shift, peak broadening or reduction of peak intensity is indicative of aggregation (see Figure 1 for compound **4 a** and Figures S25–S32 for the remaining compounds). All particles showed good stability for several hours at 25 °C and a change in the RPB is detectable after 24 h and more pronounced after 48 h. However, **4 c**, which was dispersed in water, showed a red shift of the RPB after 1 h indicating nanoparticle aggregation. This result was further confirmed through dynamic light scattering measurements, where the hydrodynamic radius was detected at around 650±200 nm indicating rapid aggregation of the nanoparticles. When the temperature is increased to 50 °C, a faster decomposition is observed and at 80 °C most particles showed significant changes to the UV/Vis spectra after 24 h. However, at lower temperatures, the stability differences in nanoparticles bearing different ligands were more apparent—nanoparticles with *N*‐cyclohexyl substituents showed higher propensity to aggregate compared to those with *N‐* ^*t*^butyl substituents. Further, particles bearing *N’*‐dodecyl substituents display a higher stability than their *N’*‐octyl counterparts. It should be noted that the observed stability of NACAuNPs is comparable or in certain cases exceeds reported thermal stabilities of NHC‐stabilized AuNPs.[^7d^, ^8d^, ^8e^, ^8g^, ^8h^] In NHCAuNPs, significantly higher thermal stability is achieved either through bidentate NHC ligands^[8e]^ or through the addition of a charged carboxylic acid group which helps to stabilize NHCAuNP in water solutions.[^8d^, ^8g^] However, AuNPs stabilized by small uncharged NHC ligands display similar^[8d]^ or lower thermal stability.^[8h]^

Next, the nanoparticle stability against exogenous thiols was investigated at 25 °C and 50 °C—we omitted the studies at 80 °C as the particles already showed significantly decreased stability at 50 °C. After exposure for 6 h only particle **4 a** showed no signs of aggregation or ripening, and this observation is in good agreement with the stability observed without exogenous thiols present. However, all particles showed degradation after 24 h and 48 h. The stability of carbene stabilized AuNPs against thiols has proven to be a general challenge and thus far only two reports have demonstrated significant improvements in stability against exogenous thiols. One example was reported by Crudden *et al*. where increased stability towards thiols was achieved by utilizing chelating NHC ligands as surfactant.^[8e]^ The second report is from our group, where water soluble histidine‐2‐ylidene stabilized gold nanoparticles displayed high stability towards glutathione.^[8g]^ Nevertheless, we have shown that NAC gold complexes can be used to prepare monodisperse NACAuNPs, in a similar fashion to NHC‐stabilized AuNPs.

In conclusion, we have demonstrated that the use of NACs to stabilize AuNPs is not only feasible, but also easily adapted from procedures used in NHCAuNP synthesis. Importantly, the use of NACs as ligands offers an attractive alternative to NHCs due to their ease of synthesis and modification, offering great flexibility in ligand design, in the continuing quest towards ever more stable AuNPs. Future work will, therefore, focus on modifying NAC gold complexes to afford multi‐dentate NACAuNP compounds.

## Conflict of interest

The authors declare no conflict of interest.

## Supporting information

As a service to our authors and readers, this journal provides supporting information supplied by the authors. Such materials are peer reviewed and may be re‐organized for online delivery, but are not copy‐edited or typeset. Technical support issues arising from supporting information (other than missing files) should be addressed to the authors.

Supplementary
