# Supplementary material for: Synthetically Versatile Nitrogen Acyclic Carbene Stabilized Gold Nanoparticles
Source: Chemistry. 2020 Nov 18;26(68):15859–62. doi: 10.1002/chem.202003679 (PMC7894353; doi:10.1002/chem.202003679)
Supplement: Supplementary file 1 — Supplementary [file CHEM-26-15859-s001.pdf]

Chemistry–A European Journal

Supporting Information

Synthetically Versatile Nitrogen Acyclic Carbene Stabilized Gold Nanoparticles

Guilherme M. D. M. Rúbio,[a] Bernhard K. Keppler,[a] Jia Min Chin,*[b] and Michael R. Reithofer*[a]

Table of Contents

Materials and Methods **3**

Synthesis of isonitrile gold precursor **4**

*t*Butyl-azaneylidyne gold (I) chloride, **1** 4

*c*Hexyl-azaneylidyne- gold (I) chloride, **2** 5

Synthesis of NAC gold chloride complexes **6**

N'-(*t*butyl)-*N*-dodecylcarbamimidoyl gold(I) chloride, **3a** 6

N'-(*t*butyl)-*N*-octylcarbamimidoyl gold(I) chloride, **3b** 7

(*N'*-(*t*butyl)-*N*,*N*-diethylcarbamimidoyl) gold(I) chloride, **3c** 8

N'-(cyclohexyl)-*N*-dodecylcarbamimidoyl gold(I) chloride, **3d** 9

N'-(cyclohexyl)-*N*-octylcarbamimidoyl gold(I) chloride, **3e** 10

Synthesis of NAC-stabilized gold nanoparticles **11**

(*N'*-(*t*butyl)-*N*-dodecylcarbamimidoyl) capped gold nanoparticles, **4a** 11

(*N'*-(*t*butyl)-*N*-octylcarbamimidoyl) capped gold nanoparticles, **4b** 12

(N'-(tbutyl)-*N,N*-diethylcarbamimidoyl) capped gold nanoparticles **4c** 13

(*N'*-(cyclohexyl)-*N*-dodecylcarbamimidoyl) capped gold nanoparticles, **4d** 14

(*N'*-(cyclohexyl)-*N*-octylcarbamimidoyl) capped gold nanoparticles, **4e** 15

^{1}H and ^{13}C NMR of synthesized compounds **16**

Fig. S 1. ^{1}H NMR spectrum of *t*butyl azaneylidyne gold (I) chloride (**1**) 16

Fig. S 2. ^{13}C NMR spectrum of *t*butyl azaneylidyne gold (I) chloride (**1**) 16

Fig. S 3. ^{1}H NMR spectrum of cyclohexyl azaneylidyne gold (I) chloride (**2**) 17

Fig. S 4. ^{13}C NMR spectrum of cyclohexyl azaneylidyne gold (I) chloride (**2**) 17

Fig. S 5. ^{1}H NMR spectrum of (*N'*-(tbutyl)-*N*-dodecylcarbamimidoyl) gold(I) chloride (**3a**) 18

Fig. S 6. ^{13}C NMR spectrum of (*N'*-(tbutyl)-*N*-dodecylcarbamimidoyl) gold(I) chloride (**3a**). (a) Full overview of ^{13}C spectrum. (b) Expanded view of the 80 ppm-0 ppm region. 19

Fig. S 7. ^{1}H NMR spectrum of (*N'*-(tbutyl)-*N*-octylcarbamimidoyl) gold(I) chloride (**3b**) 20

Fig. S 8. ^{13}C NMR spectrum of (*N'*-(*t*butyl)-*N*-octylcarbamimidoyl) gold(I) chloride (**3b**). (a) Full overview of ^{13}C spectrum. (b) Expanded view of the 80 ppm-0 ppm region. 21

Fig. S 9. ^{1}H NMR spectrum of (*N*'-(*t*butyl)-*N*,*N*-diethylcarbamimidoyl) gold(I) chloride (**3c**) 22

Fig. S 10. ^{13}C NMR spectrum of (*N*'-(*t*butyl)-*N*,*N*-diethylcarbamimidoyl) gold(I) chloride (**3c**) 23

Fig. S 11. ^{1}H NMR spectrum of (*N'*-(cyclohexyl)-*N*-dodecylcarbamimidoyl) gold(I) chloride (**3d**) .. 24

Fig. S 12. ^{13}C NMR spectrum of (*N'*-(cyclohexyl)-*N*-dodecylcarbamimidoyl) gold(I) chloride (**3d**). (a) Full overview of ^{13}C spectrum. (b) Expanded view of the 80 ppm-0 ppm region. 25

Fig. S 13. ^{1}H NMR spectrum of (*N'*-(cyclohexyl)-*N*-octylcarbamimidoyl) gold(I) chloride (**3e**) 26

Fig. S 14. ^{13}C NMR spectrum of (*N'*-(cyclohexyl)-*N*-octylcarbamimidoyl) gold(I) chloride (**3e**) 27

Fig. S 15. ^{1}H NMR spectrum of (*N'*-(tbutyl)-*N*-dodecylcarbamimidoyl) gold nanoparticles (**4a**) 28

Fig. S 16. ^{13}C NMR spectrum of (*N'*-(^{t}butyl)-*N*-dodecylcarbamimidoyl) gold nanoparticles (**4a**). (a) Full overview of ^{13}C spectrum. (b) Expanded view of the 80 ppm-0 ppm region. 29
Fig. S 17. ^{1}H NMR spectrum of (*N'*-(^{t}butyl)-*N*-octylcarbamimidoyl) gold nanoparticles (**4b**) 30
Fig. S 18. ^{13}C NMR spectrum of (*N'*-(^{t}butyl)-*N*-octylcarbamimidoyl) gold nanoparticles (**4b**) 31
Fig. S 19. ^{1}H NMR spectrum of (*N*'-(^{t}butyl)-*N*,*N*-diethylcarbamimidoyl) gold nanoparticles (**4c**) 32
Fig. S 20. ^{13}C NMR spectrum of (*N*'-(^{t}butyl)-*N*,*N*-diethylcarbamimidoyl) gold nanoparticles (**4c**) ... 33
Fig. S 21. ^{1}H NMR spectrum of (*N'*-(cyclohexyl)-*N*-dodecylcarbamimidoyl) gold nanoparticles (**4d**) 34
Fig. S 22. ^{13}C NMR spectrum of (*N'*-(cyclohexyl)-*N*-dodecylcarbamimidoyl) gold nanoparticles (**4d**) 35
Fig. S 23. ^{1}H NMR spectrum of (*N'*-(cyclohexyl)-*N*-octylcarbamimidoyl) gold nanoparticles (**4e**) .. 36
Fig. S 24. ^{13}C NMR spectrum of (*N'*-(cyclohexyl)-*N*-octylcarbamimidoyl) gold nanoparticles (**4e**). 37
UV-Vis Stability Studies **38**
Fig. S 25. UV-Vis absorption spectra of compound **4b** in toluene. a) 25 °C; b) 50 °C; c) 80 °C. ... 38
Fig. S 26. UV-Vis absorption spectra of compound **4b** in a 10 mM 1-dodecanethiol toluene solution. a) 25 °C; b) 50 °C 39
Fig. S 27. UV-Vis absorption spectra of compound **4c** in water. a) 25 °C; b) 50 °C; c) 80 °C. 40
Fig. S 28. UV-Vis absorption spectra of compound **4c** in a 10 mM GSH water solution at pH 7.4. a) 25 °C; b) 50 °C 41
Fig. S 29. UV-Vis absorption spectra of compound **4d** in toluene. a) 25 °C; b) 50 °C; c) 80 °C. ... 42
Fig. S 30. UV-Vis absorption spectra of compound **4d** in a 10 mM 1-dodecanethiol toluene solution. a) 25 °C; b) 50 °C. 43
Fig. S 31. UV-Vis absorption spectra of compound **4e** in toluene. a) 25 °C; b) 50 °C; c) 80 °C. ... 44
Fig. S 32. UV-Vis absorption spectra of compound **4e** in a 10 mM 1-dodecanethiol toluene solution. a) 25 °C; b) 50 °C. 45
Thermo-gravimetric analysis **46**
Fig. S 33. Thermogravimetric analysis of compound **4a**. 46
Fig. S 34. Thermogravimetric analysis of compound **4b.** 47
Fig. S 35. Thermogravimetric analysis of compound **4c.** 48
Fig. S 36. Thermogravimetric analysis of compound **4d**. 49
Fig. S 37. Thermogravimetric analysis of compound **4e**. 50
TEM micrographs **51**
Fig. S 38. TEM images of **4a**. 51
Fig. S 39. TEM images of **4b**. 52
Fig. S 40. TEM images of **4c**. 53
Fig. S 41. TEM images of **4d**. 54
Fig. S 42. TEM images of **4e**. 55
References **56**

Materials and Methods

All experiments were performed in air. *Cyclo*-hexyl isocyanide and *tert*-butyl isocyanide were purchased from Alfa Aesar, borane *tert*-butylamine complex, octylamine, dodecylamine and tetrahydrothiophene were purchased from Sigma Aldrich, hydrogen tetrachloroaurate was purchased from Johnson Matthey, dichloromethane, diethyl ether, ethyl acetate, tetrahydrofuran and toluene were purchased from VWR chemicals. All purchased chemicals were used as received. Chloro(tetrahydrothiophene)gold was synthesised according to literature procedure.[1]

^{1}H and ^{13}C NMR spectra were recorded at the NMR Centre, Faculty of Chemistry, University of Vienna utilizing a Bruker 700 MHz spectrometer, with TMS ∂ H = 0 ppm or residual protic solvent peak [$CDCl_3$, ∂ H = 7.26 and ∂ C=77.16; D_2O, ∂ H = 4.79 ppm] as the internal standard. Chemical shifts are given in ppm (∂) and coupling constants (*J*) are given in Hertz (Hz). Topspin 4.0.8 was used to analyse the NMR spectra. Ultraviolet-visible (UV-vis) spectroscopy was carried out using a PerkinElmer spectrophotometer Lambda 35. Samples were prepared at a concentration of 50 µg/mL in toluene or in water.

The average diameter (D) and the size distribution of the nanoparticles were determined by using ImageJ software and by measuring a minimum of 100 randomly selected nanoparticles in arbitrarily chosen areas of various obtained images. The size distribution is reported as the standard deviation (σ) which is calculated according to the following formula: $\sigma = \{(Di - D)^2/ (n - 1)\}^{1/2}$.

High resolution MS (HRMS) was measured at the Mass Spectrometry Centre, Faculty of Chemistry, University of Vienna utilizing a Bruker maXis UHR-TOF.

TEM solid samples were dispersed in 100% toluene or 100% water (when applicable). 5 µL drops of all samples were put onto carbon-coated copper grids and allowed to quick dry in an oven at 70 °C or in some cases under vacuum. Images were obtained at the Physics of Nanostructured Materials Centre, Faculty of Physics, University of Vienna, using a Gatan Digital Micrograph software with a Gatan Orius SC600 camera attached to a Philips CM200 TEM running at 200 kV.

Thermogravimetric analysis (TGA) was performed using a Mettler Toledo TGA/SDTA851^{e} instrument in the temperature range of 25 – 700 °C under N_2 atmosphere, at a heating rate of 10 $°C\cdot min^{-1}$.

Dynamic light scattering (DLS) and zeta potential measurements were performed using a Malvern Zetasizer Nano ZS at 25 °C .

Synthesis of isonitrile gold precursor

General Procedure

The reaction was carried out following a similar procedure as previously reported with slight modifications.[2] Typically, tetrahydrothiophene gold chloride (500 mg, 1.6 mmol, 1 equiv) was dissolved in 25 mL DCM and subsequently an isocyanide (*tert*-butyl isocyanide (215 µL, 1.9 mmol, 1.2 equiv) for compound **1** and *cyclo*-hexyl isocyanide (236 µL, 1.9 mmol, 1.2 equiv) for compound **2**) was added to the stirring solution. After 3 h at T~22 °C the solvent and unreacted isocyanide were removed under reduced pressure, affording a white compound. The compound was used without further purification.

***t*Butyl-azaneylidyne gold (I) chloride, 1**

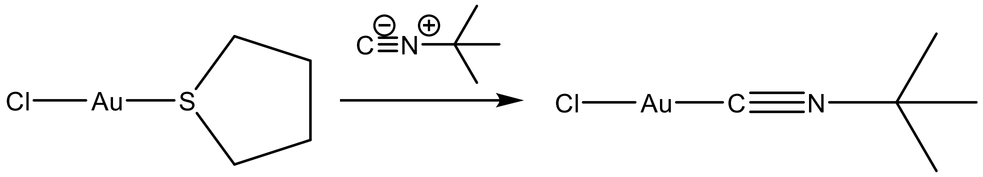

Procedure

The synthesis was carried out as described in the general procedure, using *tert*-butyl isocyanide (215 µL, 1.9 mmol, 1.2 equiv) as the reacting isocyanide. Yield: 485 mg (96%). ^{1}H NMR (700 MHz, $CDCl_3$): ∂ = 1.54 (3H, NCCH_3) ppm; ^{13}C NMR (700 MHz, $CDCl_3$): ∂= 29.7 (3C, NC(**C**H_3)$_3$), 59.2 (1C, N**C**(CH_3)$_3$), 131.9 (1C, Au**C**NC) ppm.

MS (m/z): Calcd for C_5H_9NAuCl: 315.01, Found $[C_5H_9NAuCl + Cl]^-$: 349.91.

^{c}Hexyl-azaneylidyne- gold (I) chloride, 2

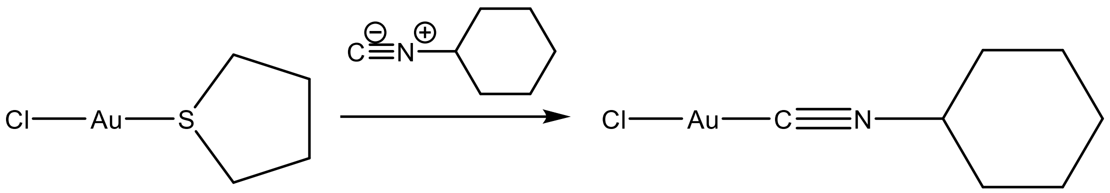

Procedure

The synthesis was carried out as described in the general procedure, using *cyclo*-hexyl isocyanide (236 µL, 1.9 mmol, 1.2 equiv) as the reacting isocyanide. Yield: 513 mg (94%). ^{1}H NMR (700 MHz, $CDCl_3$): ∂=1.41 (4H, CH(C***H***$_2$CH$_2$)$_2$); 1.72 (4H, CH(CH$_2$C***H***$_2$)$_2$); 1.96 (2H, (CH$_2$CH$_2$)$_2$C***H***$_2$); 3.89 (1H, C***H***(CH$_2$CH$_2$)$_2$) ppm; ^{13}C NMR (700 MHz, $CDCl_3$): ∂=22.6 (1C, CH(CH$_2$CH$_2$)$_2$ ***C***H$_2$), 24.5 (2C, CH(CH$_2$***C***H$_2$)$_2$ CH$_2$), 31.0 (2C, CH(***C***H$_2$CH$_2$)$_2$ CH$_2$), 55.0 (1C, ***C***H(CH$_2$CH$_2$)$_2$ CH$_2$), 133.4 (1C, Au***C***NC) ppm.

MS (m/z): Calcd for $C_7H_{11}NAuCl$: 341.02, Found $[C_7H_{11}NAuCl + Cl]^-$: 375.95.

Synthesis of NAC gold chloride complexes

<u>General Procedure</u>

The isonitrile gold chloride precursor (1 equiv), compound **1** or **2** (respectively, 450 mg, 1.4 mmol, 1 equiv or 450 mg, 1.3 mmol, 1 equiv) was dissolved in 25 mL DCM and 2 equivalents of a primary or secondary amine was then added to the reaction mixture. The reaction mixture was stirred for 24 h or 3h (respectively, when containing a primary amine or a secondary amine), and subsequently the solvent mixture was removed under reduced pressure, affording the crude compound.

***N'*-(tbutyl)-*N*-dodecylcarbamimidoyl gold(I) chloride, 3a**

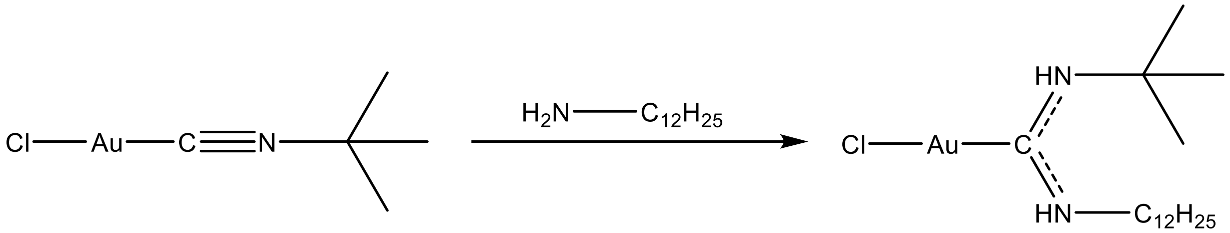

<u>Procedure</u>

The synthesis was carried out as described in the general procedure, using compound **1** as isonitrile gold chloride precursor and dodecylamine as primary amine. The reaction afforded a white powder and it was found to have sufficient purity and was used without further purification. Yield: 636 mg (91%). ^{1}H NMR (700 MHz, $CDCl_3$): ∂ = 2.61 (2H, CNHC***H*₂**CH2); 1.56 (9H, NC(C***H*₃**)3); 1.27 (20H, NCH2(C***H*₂**)10CH3); 0.88 (3H, N(CH2)11C***H*₃**) ppm. ^{13}C NMR (700MHz, $CDCl_3$): ∂ = 50.8 (1C, N***C***(CH3)3), 41.5 (1C, N***C***H2(CH2)10CH3), 32.0 (1C, NCH2(***C***H2)(CH2)9CH3), 31.4 (3C, (NC(***C***H3)3), 31.3-29.6 (7C, N(CH2)2(***C***H2)7(CH2)2CH3, 26.8 (1C, N(CH2)9***C***H2CH2CH3), 22.8 (1C, N(CH2)10***C***H2CH3, 14.2 (1C, N(CH2)11***C***H3) ppm.

MS (m/z): Calcd for $C_{17}H_{36}N_2AuCl$: 500.22, Found $[C_{17}H_{36}N_2AuCl + Na]^+$: 523.2106.

_N'_-(^{t}butyl)-_N_-octylcarbamimidoyl gold(I) chloride, 3b

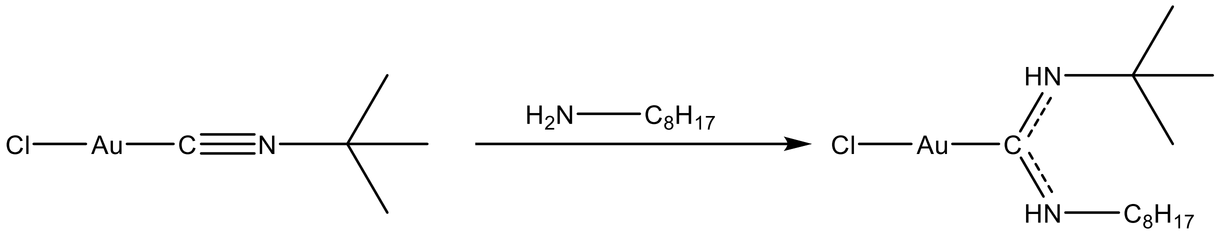

Procedure

The synthesis was carried out as described in the general procedure, using compound **1** as isonitrile gold chloride precursor and octylamine as primary amine. The reaction afforded a white powder and it was found to have sufficient purity and was used without further purification. Yield: 599 mg (96%). ^{1}H NMR (700MHz, $CDCl_3$): ∂ = 8.52-8.46 (1H, AuC(N***H***)(N***H***)); 3.52-2.99 (2H, $NC\boldsymbol{H_2}(CH_2)_6CH_3$); 1.63 (2H, $NCH_2C\boldsymbol{H_2}(CH_2)_5CH_3$); 1.56-1.49 (9H, $NC(C\boldsymbol{H_3})_3$); 1.39-1.16 (10H, $N(CH_2)_2(C\boldsymbol{H_2})_5CH_3$; 0.85 (3H, $N(CH_2)_7C\boldsymbol{H_3}$) ppm. ^{13}C NMR (700MHz, $CDCl_3$): ∂ = 191 (Au***C***(NH)(NH)); 50.6 (1C, $N\boldsymbol{C}(CH_3)_3$), 31.8 (1C, $N\boldsymbol{C}H_2(CH_2)_6CH_3$), 31.4 (3C, $(NC(\boldsymbol{C}H_3)_3)$; 29.2 (3C, $N(CH_2)(\boldsymbol{C}H_2)_3(CH_2)_3CH_3$); 27.5 (1C, $N(CH_2)_4(\boldsymbol{C}H_2)(CH_2)_2CH_3$) , 26.7 (1C, $N(CH_2)_5\boldsymbol{C}H_2CH_2CH_3$), 22.7 (1C, $N(CH_2)_6\boldsymbol{C}H_2CH_3$, 14.0 (1C, $N(CH_2)_7\boldsymbol{C}H_3$) ppm.

MS (m/z): Calcd for $C_{13}H_{28}N_2AuCl$: 444.16, Found $[C_{13}H_{28}N_2AuCl + Na]^+$: 467.1482.

(*N'*-(^{t}butyl)-*N*,*N*-diethylcarbamimidoyl) gold(I) chloride, 3c

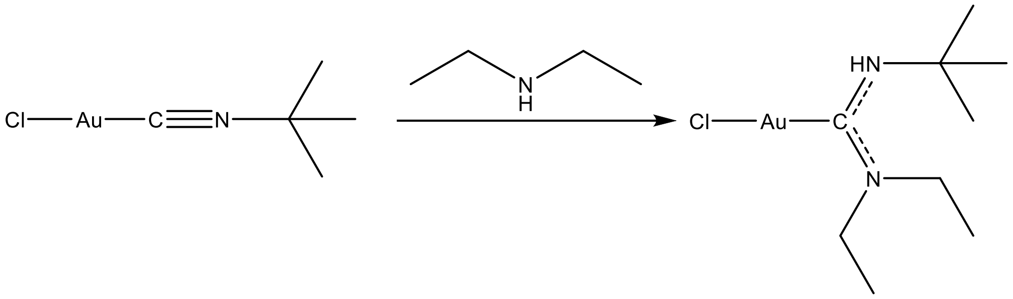

Procedure

The synthesis was carried out as described in the general procedure, using compound **1** as isonitrile gold chloride precursor and diethylamine as secondary amine. The reaction afforded a white powder which was used without further purifications. Yield: 522mg (96%). ^{1}H NMR (700MHz, $CDCl_3$): ∂ = 5.73 (1H, NCNH); 3.97 (2H, NCH_2CH_3); 3.29 (2H, NCH_2CH_3); 1.62 (9H, $NCCH_3$); 1.27 (3H, NCH_2CH_3); 1.18 (3H, NCH_2CH_3) ppm; ^{13}C NMR (700MHz, $CDCl_3$): ∂ = 189.5 (1C, AuC(NtBu)(NEt_2)); 55.3 (1C, $NC(CH_3)_3$); 40.5 (2C, CH_2CH_3); 31.8 (3C, $C(CH_3)_3$); 14.8 (1C, CH_2CH_3); 11.9 (1C, CH_2CH_3) ppm.
MS (m/z): Calcd for $C_9H_{20}N_2AuCl$: 388.10, Found $[C_9H_{20}N_2AuCl + Na]^+$: 411.0859.

***N'*-(cyclohexyl)-*N*-dodecylcarbamimidoyl gold(I) chloride, 3d**

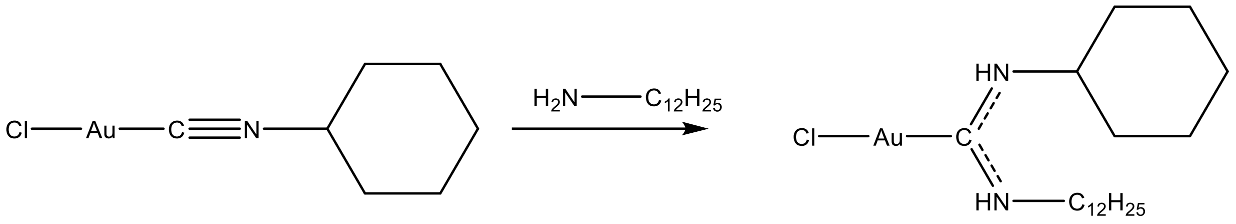

<u>Procedure</u>

The synthesis was carried out as described in the general procedure, using compound **2** as isonitrile gold chloride precursor and dodecylamine as primary amine. The reaction afforded a whitish oil which was used without further purification. Yield: 651mg (95%). ^{1}H NMR (700MHz, $CDCl_3$): ∂ =8.51-8.45 (1H, $AuC(N\boldsymbol{H})(N\boldsymbol{H})$); 3.69 (1H, $NC\boldsymbol{H}(CH_2)(CH_2)$); 3.45 (2H, $(C\boldsymbol{H_2})(CH_2)_{10}CH_3$); 1.9 (2H, $(CH_2)(C\boldsymbol{H_2})(CH_2)_9CH_3$); 1.72 (2H, arom. $C\boldsymbol{H_2}(CH_2)(CH_2)$); 1.54 (2H, $(CH_2)_2(C\boldsymbol{H_2})(CH_2)_8CH_3$); 1.09-1.38 (26H, 8H arom+16H, $(CH_2)_3(C\boldsymbol{H_2})_{\boldsymbol{8}}CH_3$)); 0.8 (3H, $CH_2C\boldsymbol{H_3}$) ppm. ^{13}C NMR (700MHz, $CDCl_3$): ∂= 57.7 (1C, $N\boldsymbol{C}(CH_2)(CH_2)$); 49.8 (1C, $\boldsymbol{C}H_2(CH_2)_{10}CH_3$); 48.9 (2C, $CH_2(\boldsymbol{C}H_2)_2(CH_2)_8CH_3$); 34.2 (2C, $NC(\boldsymbol{C}H_2)_2(CH_2)_2CH_2$); 32.0 (1C, $(CH_2)_3(\boldsymbol{C}H_2)(CH_2)_7CH_3$); 31.5 (1C, $(CH_2)_4(\boldsymbol{C}H_2)(CH_2)_6CH_3$); 29.6 (3C, $(CH_2)_4(\boldsymbol{C}H_2)_3(CH_2)_4CH_3$); 29.5 (1C, $(CH_2)_7(\boldsymbol{C}H_2)(CH_2)_3CH_3$); 29.4 (1C, $(CH_2)_8(\boldsymbol{C}H_2)(CH_2)_2CH_3$); 26.8 (1C, $NC(CH_2)(CH_2)\boldsymbol{C}H_2$)); 24.9 (2C, $NC(CH_2)_2(\boldsymbol{C}H_2)_2CH_2$); 22.7 (1C, $(CH_2)_{10}(\boldsymbol{C}H_2)CH_3$); 14.1 (1C, $(CH_2)_{11}\boldsymbol{C}H_3$) ppm.

MS (m/z): Calcd for $C_{19}H_{38}N_2AuCl$: 526.24, Found $[C_{19}H_{38}N_2AuCl + Na]^+$: 549.2256.

***N'*-(cyclohexyl)-*N*-octylcarbamimidoyl gold(I) chloride, 3e**

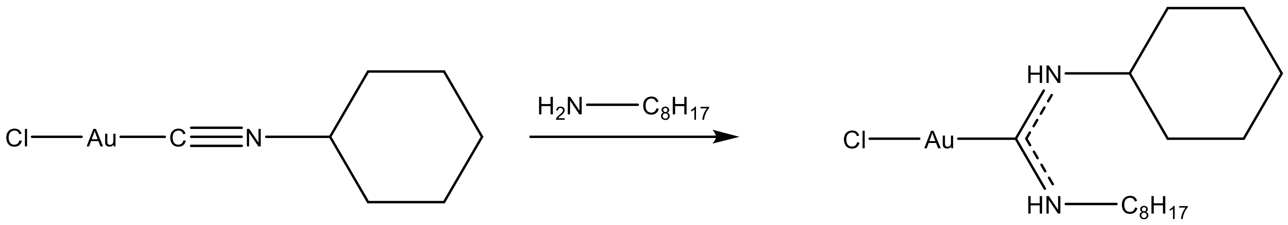

<u>Procedure</u>

The synthesis was carried out as described in the general procedure, using compound **2** as isonitrile gold chloride precursor and octylamine as primary amine. The reaction afforded white powder which was used without further purification. Yield: 651 mg (95%). ^{1}H NMR (700MHz, $CDCl_3$): ∂ = 8.41-8.36 (1H, AuC(N*H*)(N*H*)); 3.7 (1H, $NC\boldsymbol{H}(CH_2)(CH_2)$); 3.4 (2H, $(C\boldsymbol{H_2})(CH_2)_6CH_3$); 1.94 (2H, $(CH_2)(C\boldsymbol{H_2})(CH_2)_5CH_3$); 1.74 (2H, $C\boldsymbol{H_2}(CH_2)_2(CH_2)_2CHN$); 1.56 (2H, $(CH_2)_2(C\boldsymbol{H_2})(CH_2)_4CH_3$); 1.09-1.45 (14H, (8H, $CH_2(C\boldsymbol{H_2})_2(C\boldsymbol{H_2})_2CHN$) and (6H, $(CH_2)_4(C\boldsymbol{H_2})_3CH_3$)); 0.8 (3H, $CH_2C\boldsymbol{H_3}$); ^{13}C NMR (700MHz, $CDCl_3$): ∂ = 190.7 (1C, Cl-Au-$\boldsymbol{C}$-$(NCH_2)(NCH)$); 57.8 (1C, $N\boldsymbol{C}(CH_2)(CH_2)$); 49.0 (1C, $(\boldsymbol{C}H_2)(CH_2)_6CH_3$); 34.2 (2C, $NC(\boldsymbol{C}H_2)_2(CH_2)_2CH_2$); 31.5 (2C, $(CH_2)(\boldsymbol{C}H_2)_2(CH_2)_4CH_3$); 29.4 (2C, $NC(CH_2)_2(\boldsymbol{C}H_2)_2CH_2$); 26.8 (1C, $NC(CH_2)_2(CH_2)_2\boldsymbol{C}H_2$); 24.9 (3C, $(CH_2)_3(\boldsymbol{C}H_2)_3(CH_2)CH_3$); 22.8 (1C, $(CH_2)_6(\boldsymbol{C}H_2)CH_3$); 14.1 (1C, $(CH_2)_7\boldsymbol{C}H_3$).

MS (m/z): Calcd for $C_{15}H_{30}N_2AuCl$: 470.18, Found $[C_{15}H_{30}N_2AuCl - H^+]^-$: 469.1693 and $[C_{15}H_{30}N_2AuCl + Cl^-]^-$: 505.1462.

Synthesis of NAC-stabilized gold nanoparticles

General Procedure

The synthesis of gold nanoparticles follows a reported procedure[3] with slight modifications.

A NAC gold chloride complex (0.1 mmol, 1 equiv) is dissolved in 5 mL THF and stirred at 1200 rpm, followed by the quick addition of $^{t}BuNH_2{\cdot}BH_3$ (31 mg, 0.35 mmol, 3.5 equiv) pre-dissolved in 50 0µL THF. The solution was stirred for 4 hours, after which 1 drop of water (~30 µL) was added to quench the borane complex allowing the mixture to stir for an additional hour. To clean the particles, the mixture was concentrated under reduced pressure, followed by the addition of ethyl acetate which allowed the centrifugation of the particles (15000 rpm). Rewashing them multiple times in ethyl acetate followed by centrifugation and drying afforded a dried dark compound that is dispersible in most non-polar organic solvents and THF.

(*N'*-(^{t}butyl)-*N*-dodecylcarbamimidoyl) capped gold nanoparticles, 4a

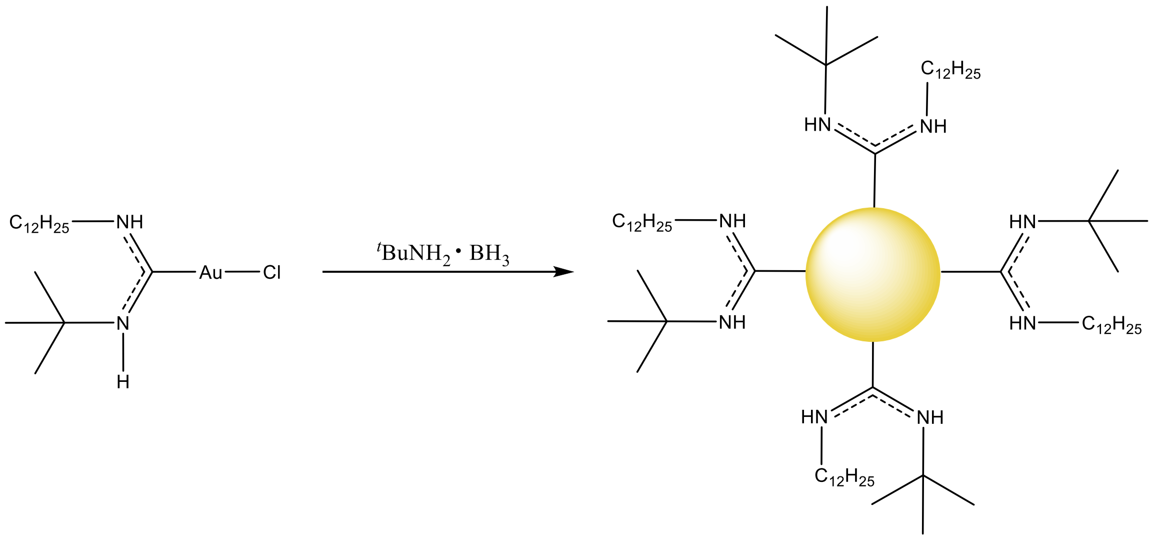

Procedure

The synthesis was carried out as described in the general procedure, using compound **3a** (50 mg, 0.1 mmol, 1 equiv). The obtained nanoparticles can be stored for several month without observable decomposition. ^{1}H NMR (700 MHz, $CDCl_3$): ∂= 1.72-1.33 (9H,$NC(C\boldsymbol{H_3})_3$); 1.47 (2H, $N(CH_2)(CH_2)_{10}CH_3$); 1.42 (2H, $N(CH_2)(CH_2)(CH_2)_9CH_3$); 1.34-1.20 (18H, $N(CH_2)_2(C\boldsymbol{H_2})_9CH_3$); 0.88 (3H, $N(CH_2)_{11}C\boldsymbol{H_3}$). ^{13}C NMR (700 MHz, $CDCl_3$): ∂ = 53.5 (1C, $N\boldsymbol{C}(CH_3)_3$), 31.9 (1C, $N\boldsymbol{C}H2(CH2)_{10}CH_3$), 29.7 (1C, $NCH_2(\boldsymbol{C}H_2)(CH_2)_9CH_3$), 29.6-29.3 (4C, $N(CH_2)_2(\boldsymbol{C}H_2)_4(CH_2)_5CH_3$, 29.3 (1C, $N(CH_2)_6\boldsymbol{C}H_2(CH_2)_4CH_3$), 29.2 (1C, $N(CH_2)_7(\boldsymbol{C}H_2)(CH_2)_3CH_3$, 27.3 (3C, $(NC(\boldsymbol{C}H3)3)$, 27.1 (1C, $N(CH_2)_8(\boldsymbol{C}H_2)(CH_2)_2CH_3$, 26.9 (1C, $N(CH_2)_9(\boldsymbol{C}H_2)(CH_2)CH_3$, 22.7 (1C, $N(CH_2)_{10}(\boldsymbol{C}H_2)CH_3$, 14.1 (1C, $N(CH_2)_{11}\boldsymbol{C}H_3$) ppm.

UV/Vis (DCM): λ_{max} = 517 nm.

(N'-(^{t}butyl)-N-octylcarbamimidoyl) capped gold nanoparticles, 4b

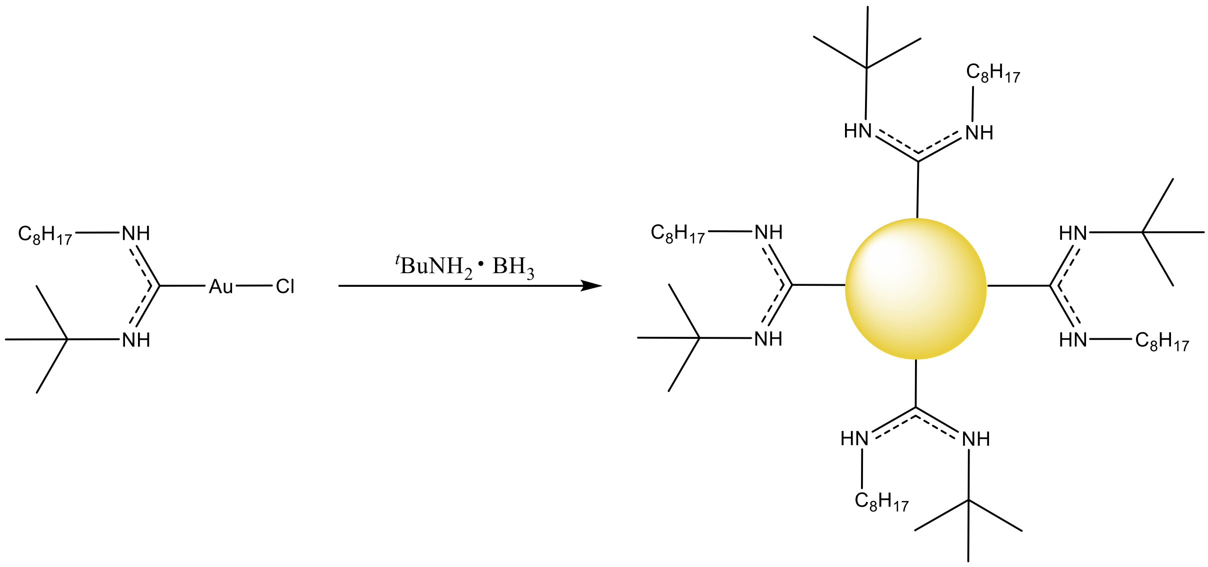

<u>Procedure</u>

The synthesis was carried out as described in the general procedure, using compound **3b** (44 mg, 0.1 mmol, 1 equiv). The obtained nanoparticles can be stored for several month without observable decomposition. ^{1}H NMR (700 MHz, $CDCl_3$): ∂ = 8.82-8.74 (1H, AuC(N***H***)(N***H***); 3.52 (2H, $NC\boldsymbol{H_2}(CH_2)_6CH_3$); 1.48 (9H, $NC(C\boldsymbol{H_3})_3$); 1.34-1.18 (10H, $NCH_2(C\boldsymbol{H_2})_5CH_3$; 0.81 (3H, $N(CH_2)_7C\boldsymbol{H_3}$); ^{13}C NMR (700 MHz, $CDCl_3$): ∂ = 206.3 (1C, Au***C***$(NC(CH_3)_3)$ $(N(C_8H_{17}))$; 52.5 (1C, N***C***$(CH_3)_3$); 31.9 (1C, N***C***$H_2(CH_2)_6CH_3$); 31.6 (3C, NC(***C***$H_3)_3$); 31.5 (1C, $N(CH_2)$(***C***$H_2)(CH_2)_5CH_3$); 29.5 (1C, $N(CH_2)_2$(***C***$H_2)(CH_2)_4CH_3$; 29.2 (2C, $N(CH_2)_3$(***C***$H_2)_2(CH_2)_2CH_3$); 26.9 (1C, $N(CH_2)_5$(***C***$H_2)(CH_2)$ CH_3); 22.8 (1C, $N(CH_2)_6$(***C***$H_2)CH_3$); 14.4 (1C, $N(CH_2)_7$***C***H_3).

UV/Vis (DCM): λ_{max} = 522 nm.

(N'-(^{t}butyl)-*N,N*-diethylcarbamimidoyl) capped gold nanoparticles 4c

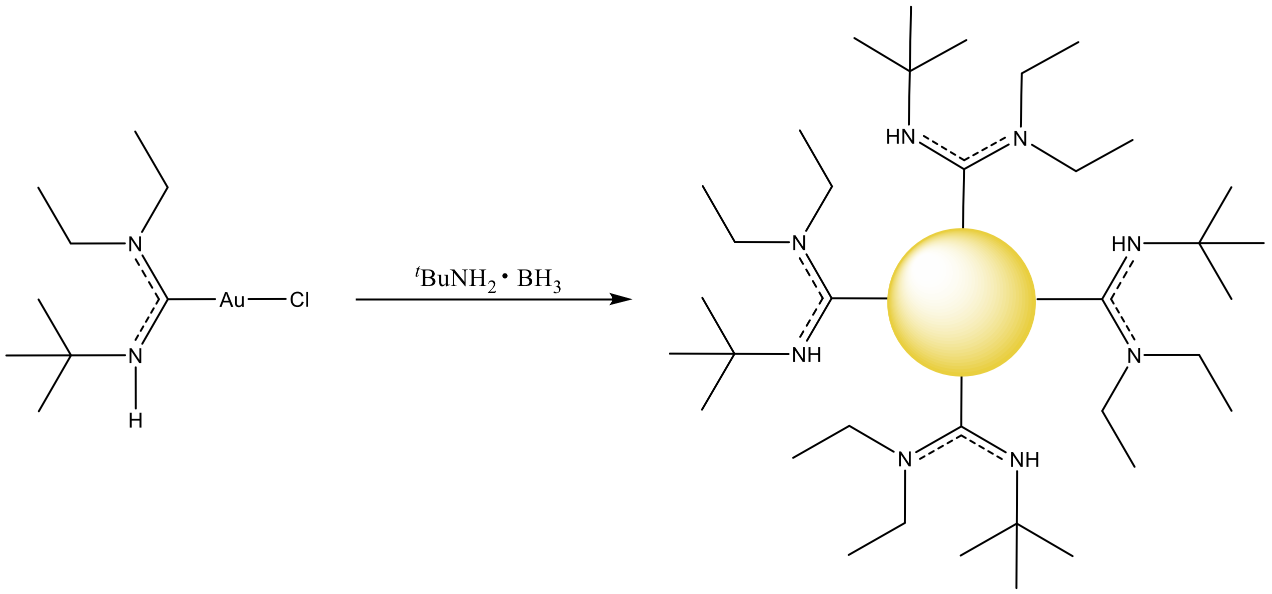

<u>Procedure</u>

The synthesis was carried out with some modifications from the general procedure. Compound **3c** (39 mg, 0.1 mmol, 1 equiv) was reduced affording water dispersible nanoparticles. In order to clean **4c**, the borane complex was quenched by the addition of a drop of water. Excess THF was added and the reaction mixture was centrifuged at 15000 rpm. The resulting pellet was resuspended in THF centrifuged several times; stable nanoparticles are obtained after freeze drying of **4c**.

^{1}H NMR (700 MHz, D_2O): ∂ = 5.80 (1H, NCN$\boldsymbol{H}$); 3.92 (2H, NC$\boldsymbol{H_2}$CH$_3$); 3.44 (2H, NC$\boldsymbol{H_2}$CH$_3$); 1.58 (9H, NC(C$\boldsymbol{H_3}$)$_3$); 1.23 (3H, NCH$_2$C$\boldsymbol{H_3}$); 1.15 (3H, NCH$_2$C$\boldsymbol{H_3}$); ^{13}C NMR (700 MHz, D_2O): ∂= 200.3 (1C, ClAuC(NC(CH$_3$)$_3$)(N(CH$_2$CH$_3$)$_2$); 55.2 (1C, AuC($\boldsymbol{C}$H$_2$CH$_3$)); 54.2 (1C, AuC($\boldsymbol{C}$H$_2$CH$_3$)); 40.3 (1C, AuC(N$\boldsymbol{C}$(CH$_3$)$_3$); 31.8 (3C, AuC(NC($\boldsymbol{C}$H$_3$)$_3$); 14.7 (1C, AuC(CH$_2$$\boldsymbol{C}H_3$)); 11.8 (1C, AuC(CH$_2$$\boldsymbol{C}H_3$)).

UV/Vis (water): λ_{max}= 523 nm.

(*N'*-(cyclohexyl)-*N*-dodecylcarbamimidoyl) capped gold nanoparticles, 4d

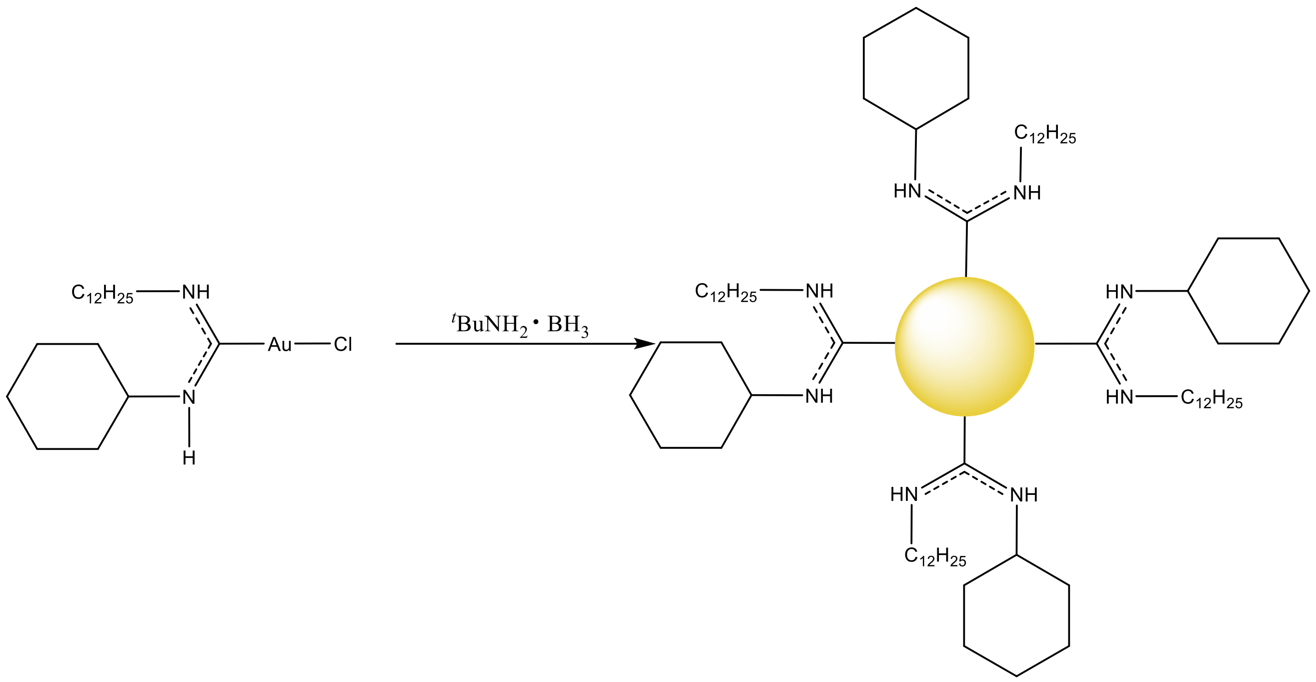

Procedure

The synthesis was carried out as described in the general procedure, using compound **3c** (53 mg, 0.1mmol, 1 equiv) as the NAC gold chloride complex. The obtained nanoparticles can be stored in the fridge (T~ 4 °C) for several months without observable decomposition.

^{1}H NMR (700 MHz, $CDCl_3$): ∂ = 3.45 (2H, N(C***H*₂**)(CH₂)₁₀CH₃); 3.17 (1H, NC***H***(CH₂)₂(CH₂)₂CH₂); 1.95 (2H, NCH(CH₂)₂(CH₂)₂C***H*₂**); 1.75 (2H, N(CH₂)(C***H*₂**)(CH₂)₉CH₃); 1.57 (4H, NCH(C***H*₂**)₂(CH₂)₂CH₂); 1.46-1.04 (22H, (4H, NCH(CH₂)₂(C***H*₂**)₂CH₂) and (18H, (C***H*₂**)₉); 0.86 (3H, N(CH₂)₁₁C***H*₃**). ^{13}C NMR (700 MHz, $CDCl_3$): ∂ = 205.9 (1C, Au***C***(NCH₂)(NCH)); 57.7 (1C, N***C***(CH₂)(CH₂)); 49.8 (1C, ***C***H₂(CH₂)₁₀CH₃); 48.9 (1C, CH₂(***C***H₂)(CH₂)₉CH₃); 34.4 (1C, (CH₂)₂(***C***H₂)(CH₂)₈CH₃); 34.0 (2C, NC(***C***H₂)₂(CH₂)₂CH₂); 32.0 (1C, (CH₂)₃(***C***H₂)(CH₂)₇CH₃); 31.5 (1C, (CH₂)₄(***C***H₂)(CH₂)₆CH₃); 29.7 (1C, (CH₂)₅(***C***H₂)(CH₂)₅CH₃); 29.6 (1C, (CH₂)₆(***C***H₂)(CH₂)₄CH₃); 29.5 (1C, (CH₂)₇(***C***H₂)(CH₂)₃CH₃); 29.4 (1C, (CH₂)₈(***C***H₂)(CH₂)₂CH₃); 29.3 (1C, (CH₂)₉(CH₂)(***C***H₂)CH₃); 26.8 (1C, NC(CH₂)(CH₂)***C***H₂)); 24.9 (2C, NC(CH₂)₂(***C***H₂)₂CH₂); 22.7 (1C, (CH₂)₁₀(***C***H₂)CH₃); 14.1 (1C, (CH₂)₁₁***C***H₃).

UV/Vis (DCM): λ_{max}=520 nm.

(*N'*-(cyclohexyl)-*N*-octylcarbamimidoyl) capped gold nanoparticles, 4e

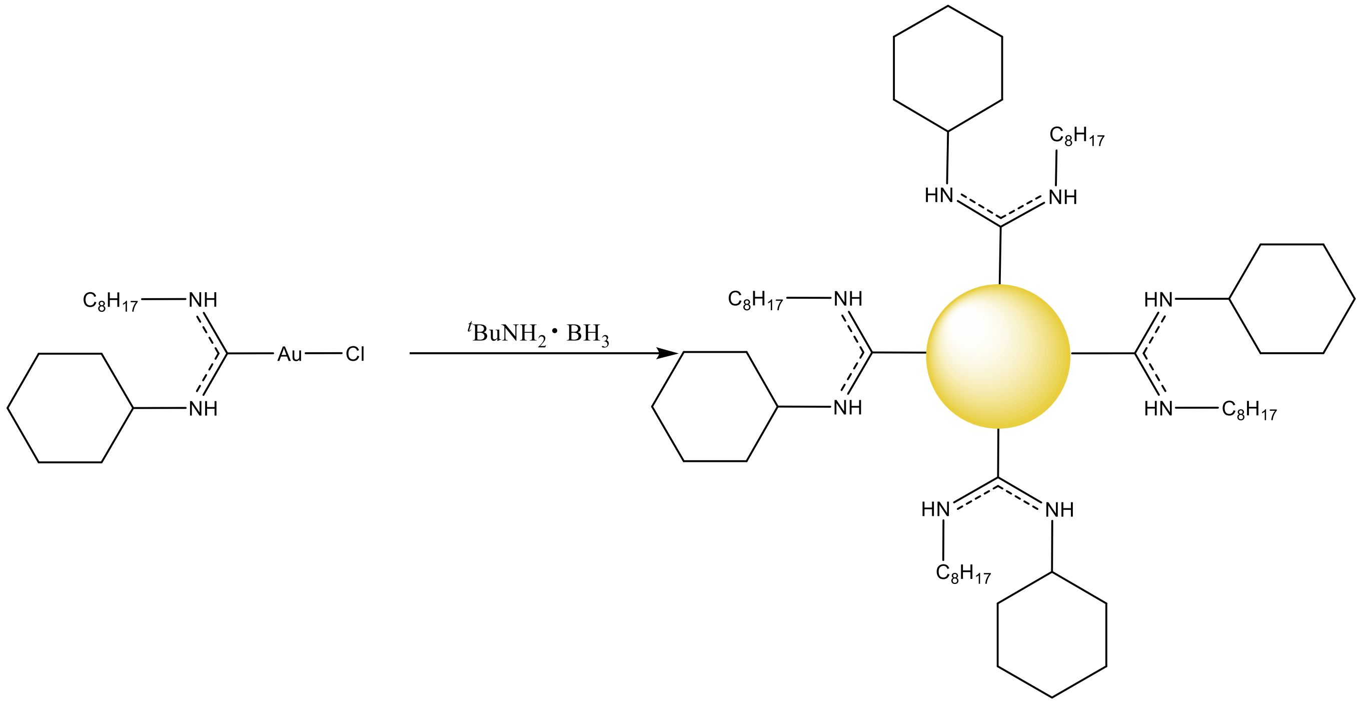

<u>Procedure</u>

The synthesis was carried out as described in the general procedure, using compound **3d** (47 mg, 0.1 mmol, 1 equiv) as the NAC gold chloride complex. The obtained nanoparticles can be stored in the fridge (T~4 °C) for several months without observable decomposition. ^{1}H NMR (700 MHz, $CDCl_3$): ∂= 3.71-3.43 (2H, $NH(C\boldsymbol{H}_2)(CH_2)_6CH_3$); 3.29 (1H, $NC\boldsymbol{H}(CH_2)(CH_2)$); 1.93 (2H, $C\boldsymbol{H}_2(CH_2)_2(CH_2)_2CHN$); 1.74 (2H, $(CH_2)(C\boldsymbol{H}_2)(CH_2)_5CH_3$); 1.55 (2H, $(CH_2)_2(C\boldsymbol{H}_2)(CH_2)_4CH_3$); 1.38 (2H, $(CH_2)_3(C\boldsymbol{H}_2)(CH_2)_3CH_3$); 1.34-1.13 (14H, (8H, $CH_2(C\boldsymbol{H}_2)_2(C\boldsymbol{H}_2)_2CHN$) and (6H, $(CH_2)_4(C\boldsymbol{H}_2)_3CH_3$))); 0.84 (3H, $CH_2C\boldsymbol{H}_3$); ^{13}C NMR (700 MHz, $CDCl_3$): ∂= 206.0 (1C, $Au\boldsymbol{C}(NCH_2)(NCH)$); 57.7 (1C, $N\boldsymbol{C}(CH_2)(CH_2)$); 48.9 (1C, $\boldsymbol{C}H_2(CH_2)_6CH_3$); 34.3 (1C, $CH_2(\boldsymbol{C}H_2)(CH_2)_5CH_3$); 31.9 (1C, $(CH_2)_2(\boldsymbol{C}H_2)(CH_2)_4CH_3$); 31.6 (2C, $NC(CH_2)(\boldsymbol{C}H_2)CH_2$); 29.5 (1C, $(CH_2)_3(\boldsymbol{C}H_2)(CH_2)_3CH_3$); 29.4 (1C, $(CH_2)_4(\boldsymbol{C}H_2)(CH_2)_2CH_3$); 26.9 (1C, $(CH_2)_5(\boldsymbol{C}H_2)(CH_2)CH_3$); 25.3 (1C, $NC(CH_2)(CH_2)\boldsymbol{C}H_2$); 22.7 (1C, $(CH_2)_6(\boldsymbol{C}H_2)CH_3$); 14.2 (1C, $(CH_2)_7\boldsymbol{C}H_3$).

UV/Vis (DCM): λ_{max}=525 nm.

^{1}H and ^{13}C NMR of synthesized compounds

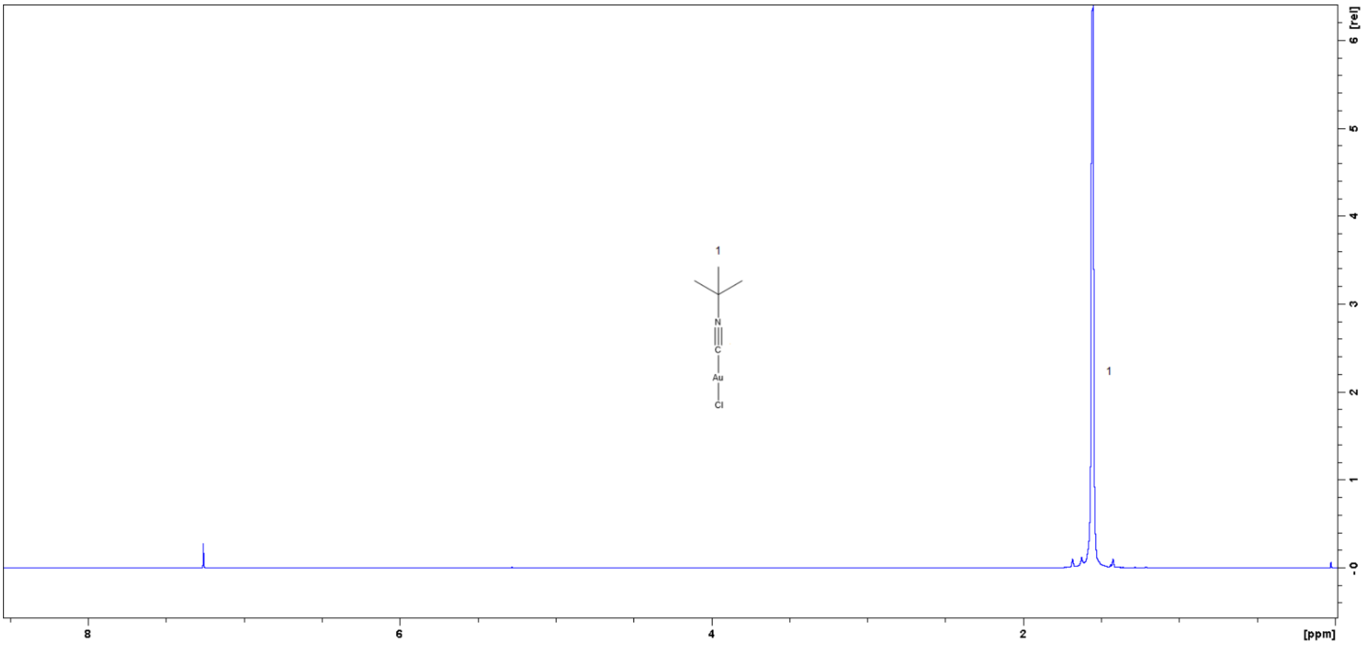

Fig. S 1. ^{1}H NMR spectrum of ^{t}butyl azaneylidyne gold (I) chloride (**1**)

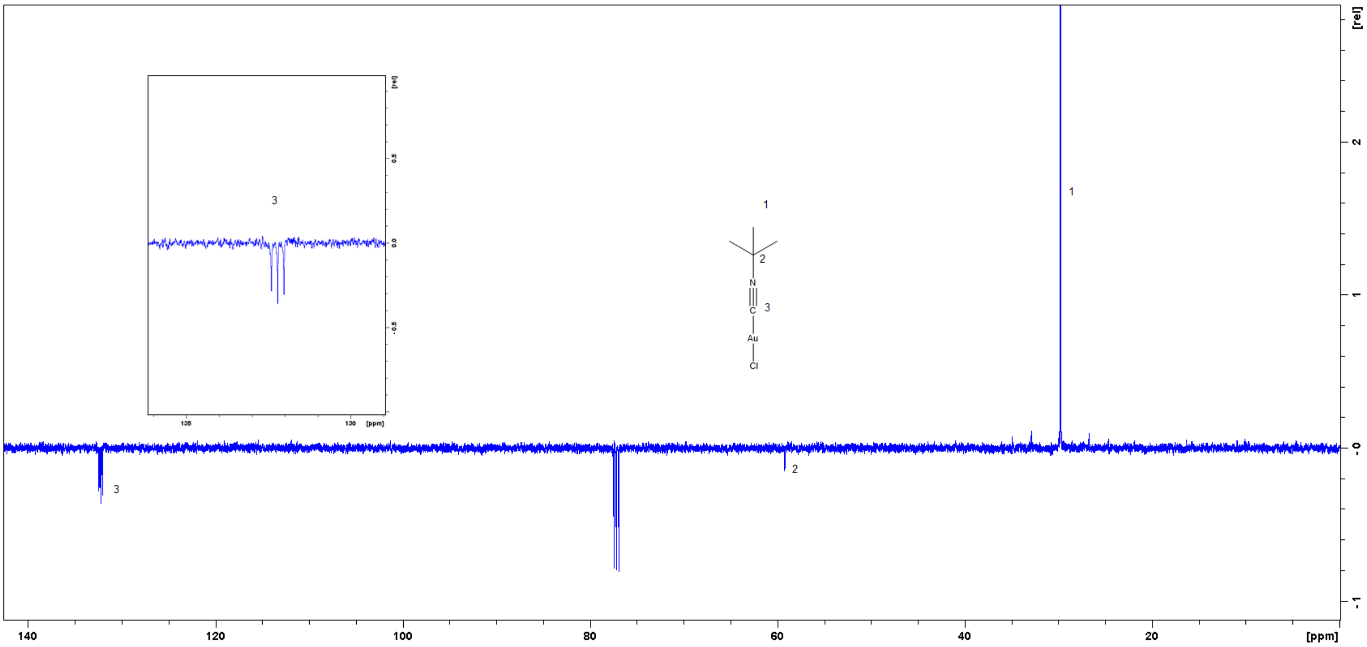

Fig. S 2. ^{13}C NMR spectrum of ^{t}butyl azaneylidyne gold (I) chloride (**1**)

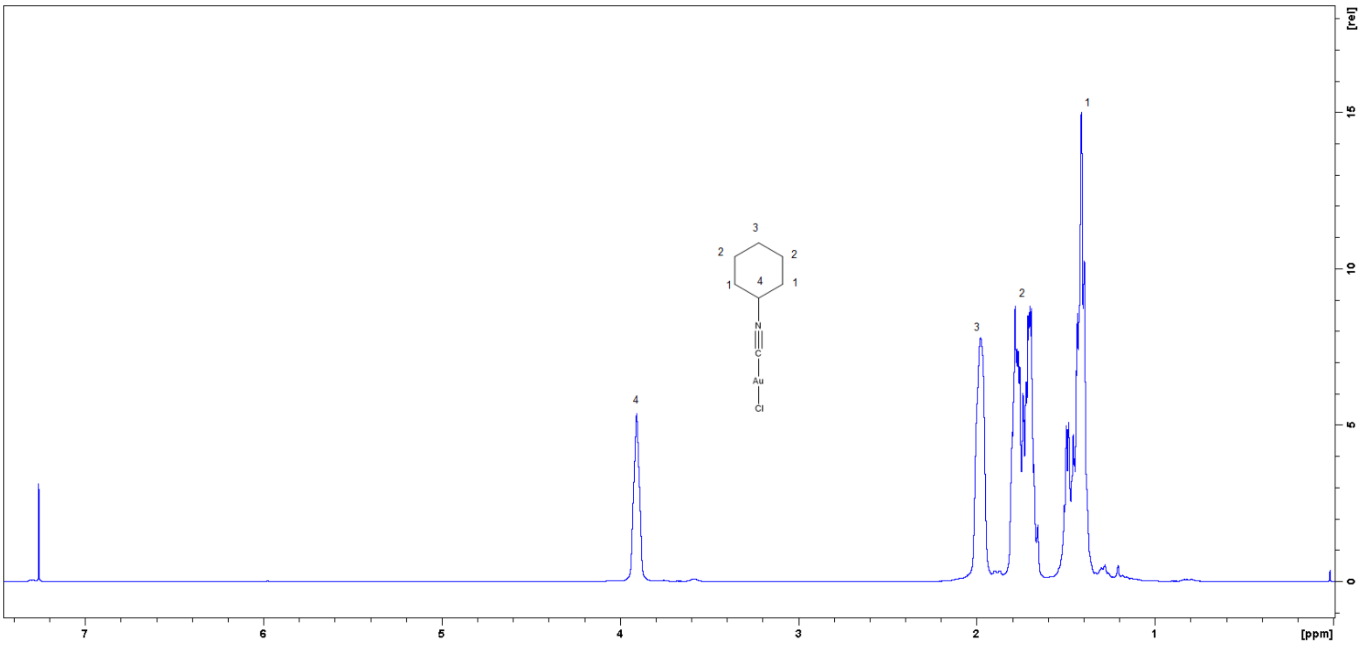

Fig. S 3. ^{1}H NMR spectrum of cyclohexyl azaneylidyne gold (I) chloride (**2**)

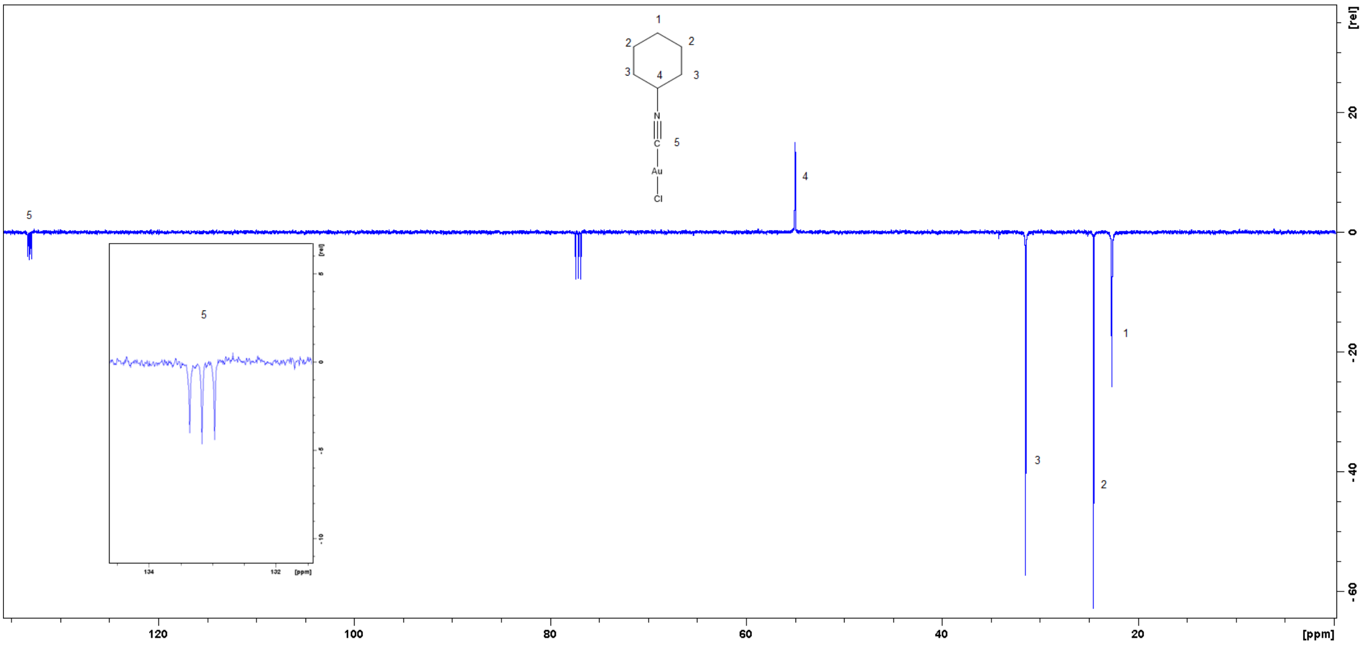

Fig. S 4. ^{13}C NMR spectrum of cyclohexyl azaneylidyne gold (I) chloride (**2**)

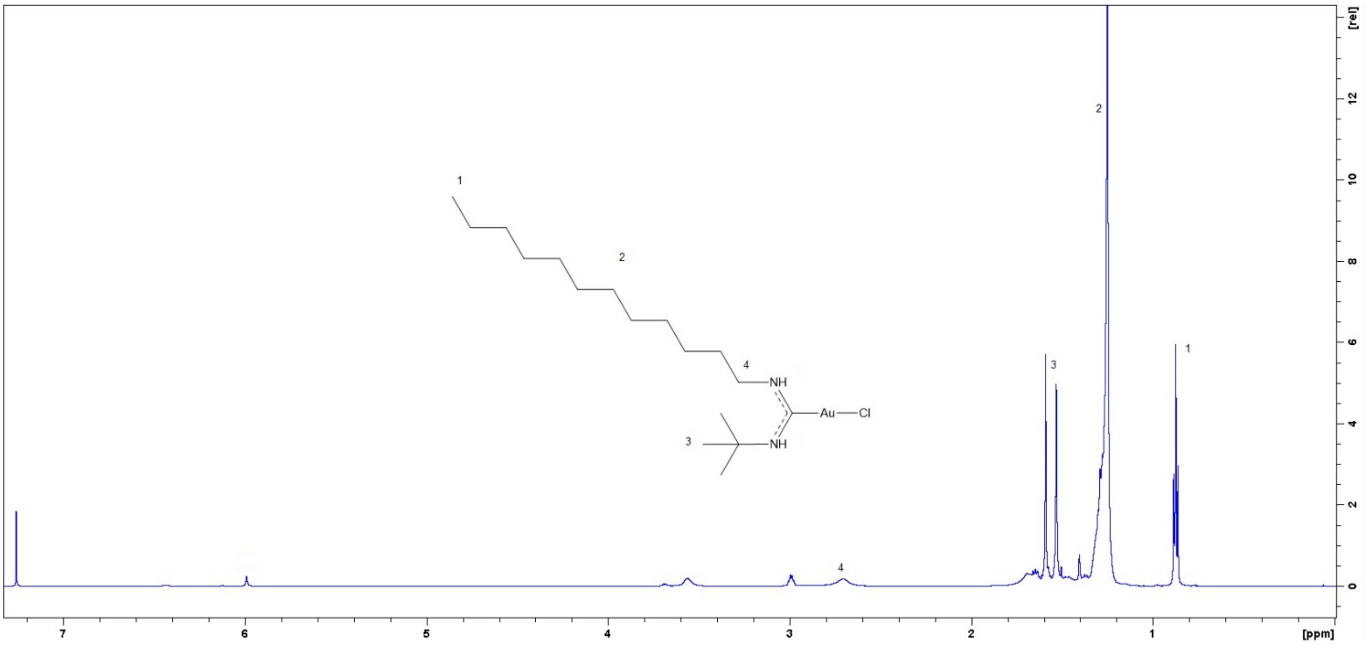

Fig. S 5. ^{1}H NMR spectrum of (*N'*-(^{t}butyl)-*N*-dodecylcarbamimidoyl) gold(I) chloride (**3a**)

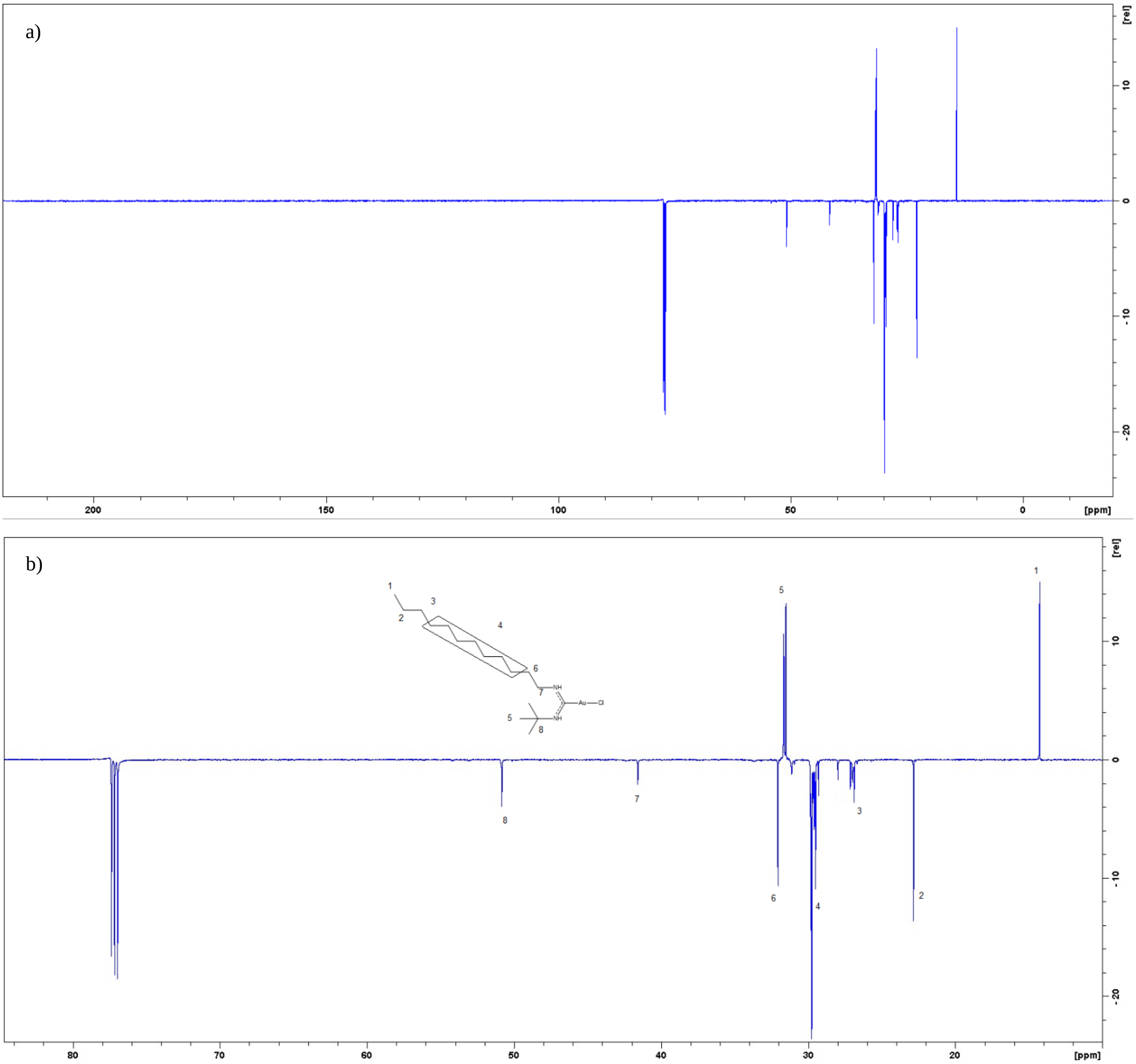

Fig. S 6. ^{13}C NMR spectrum of (*N'*-(^{t}butyl)-*N*-dodecylcarbamimidoyl) gold(I) chloride (**3a**). (a) Full overview of ^{13}C spectrum. (b) Expanded view of the 80 ppm-0 ppm region.

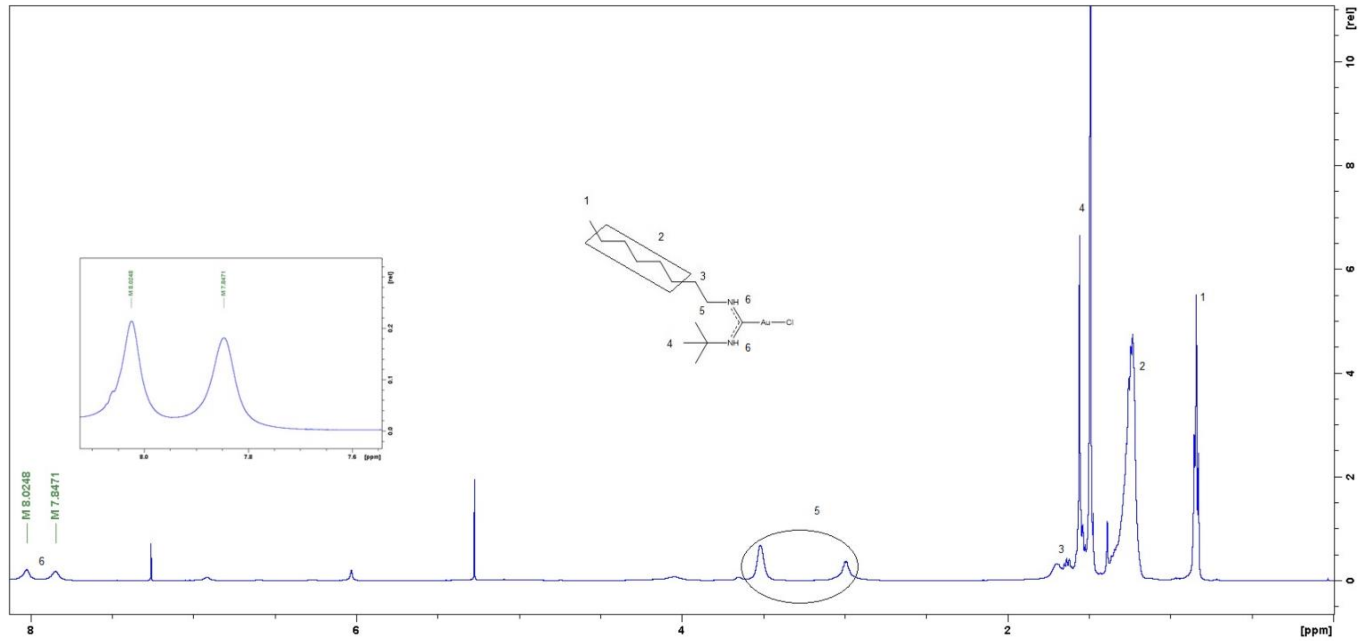

Fig. S 7. ^{1}H NMR spectrum of (*N'*-(^{t}butyl)-*N*-octylcarbamimidoyl) gold(I) chloride (**3b**)

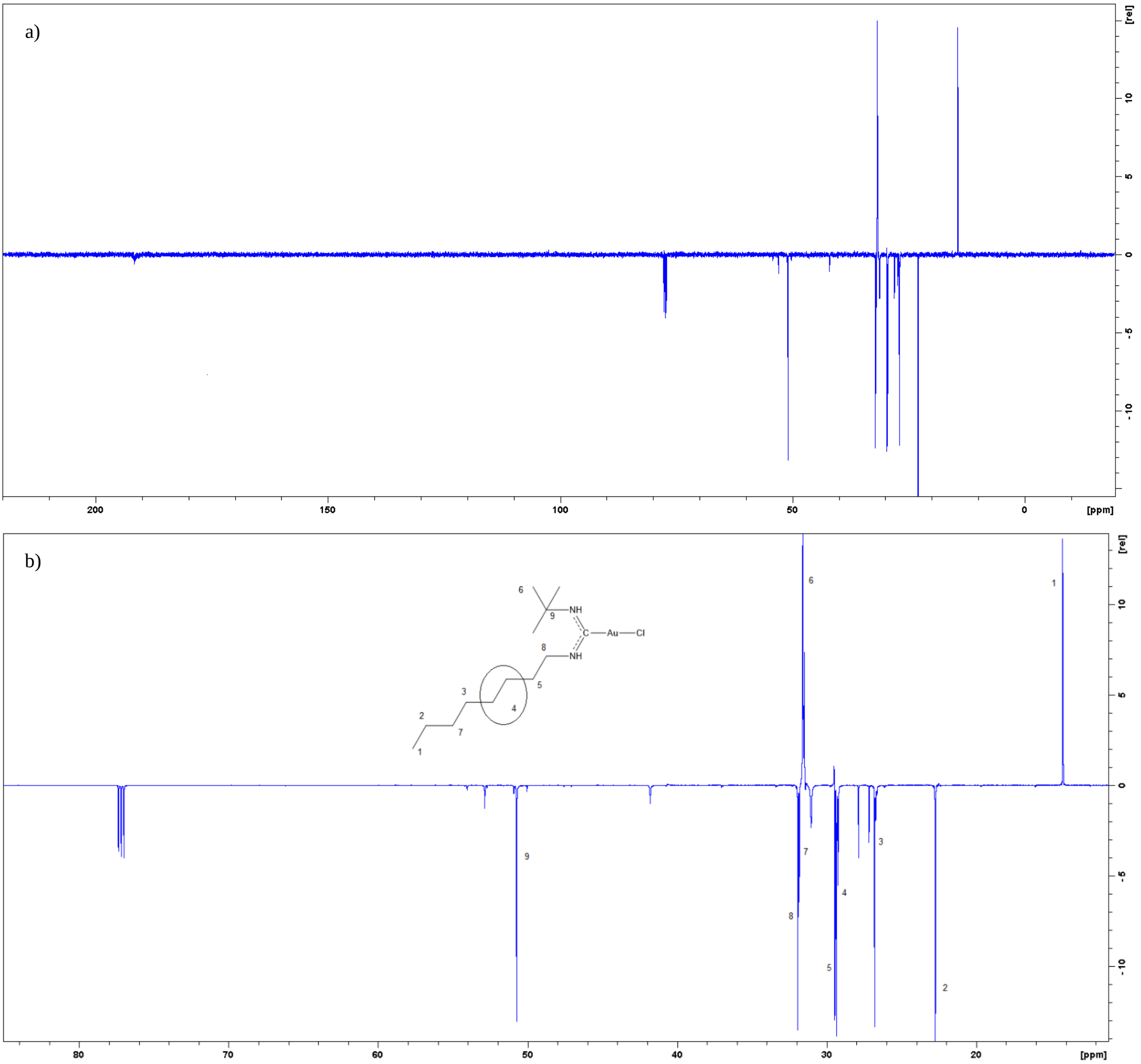

Fig. S 8. ^{13}C NMR spectrum of (*N'*-(^{t}butyl)-*N*-octylcarbamimidoyl) gold(I) chloride (**3b**). (a) Full overview of ^{13}C spectrum. (b) Expanded view of the 80 ppm-0 ppm region.

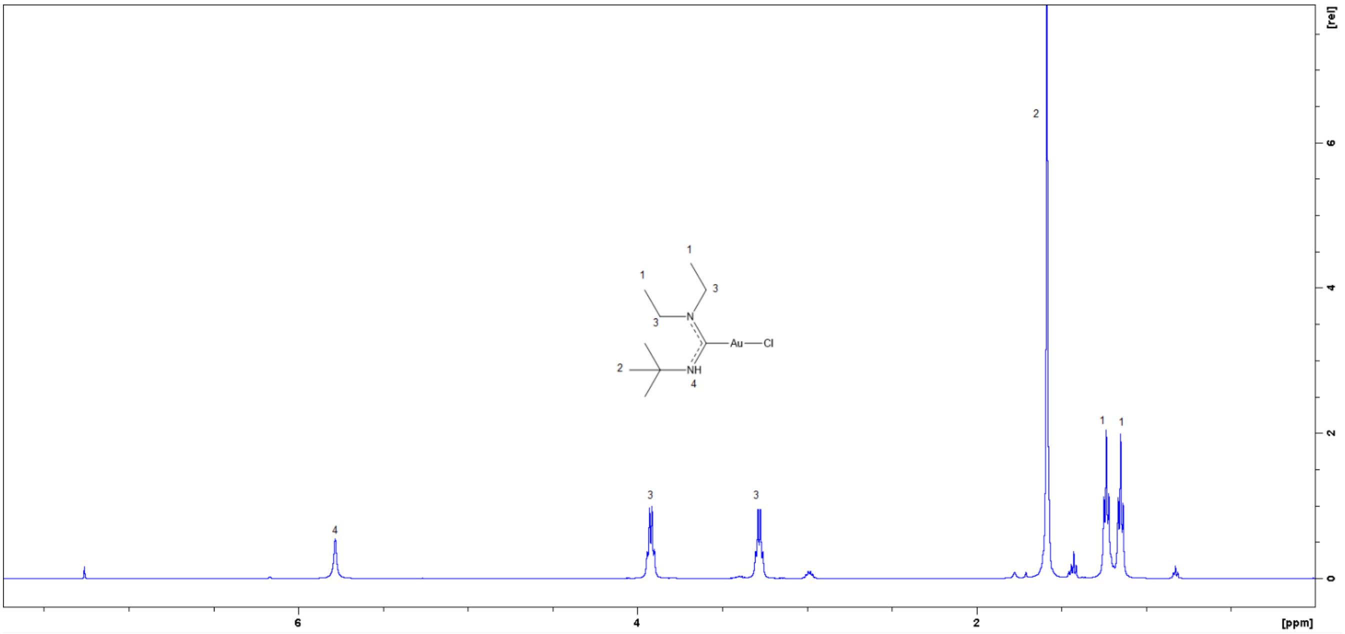

Fig. S 9. ^{1}H NMR spectrum of (*N*'-(^{t}butyl)-*N*,*N*-diethylcarbamimidoyl) gold(I) chloride (**3c**)

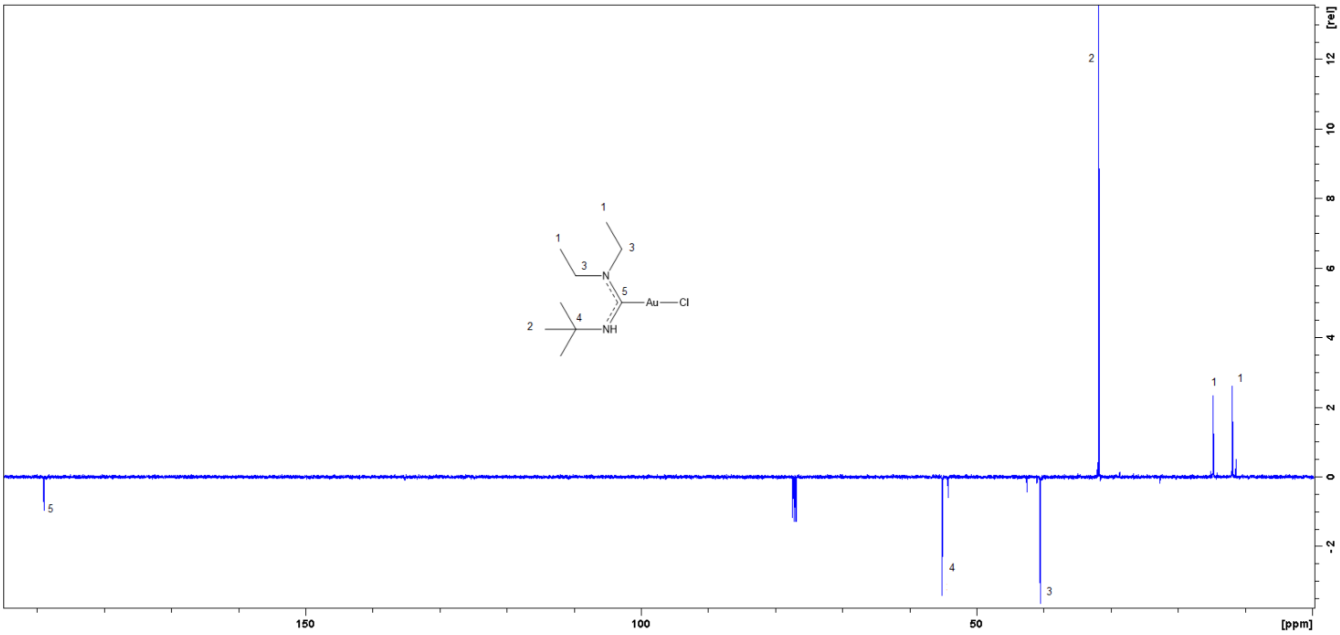

Fig. S 10. ^{13}C NMR spectrum of (*N*'-(^{t}butyl)-*N*,*N*-diethylcarbamimidoyl) gold(I) chloride (**3c**)

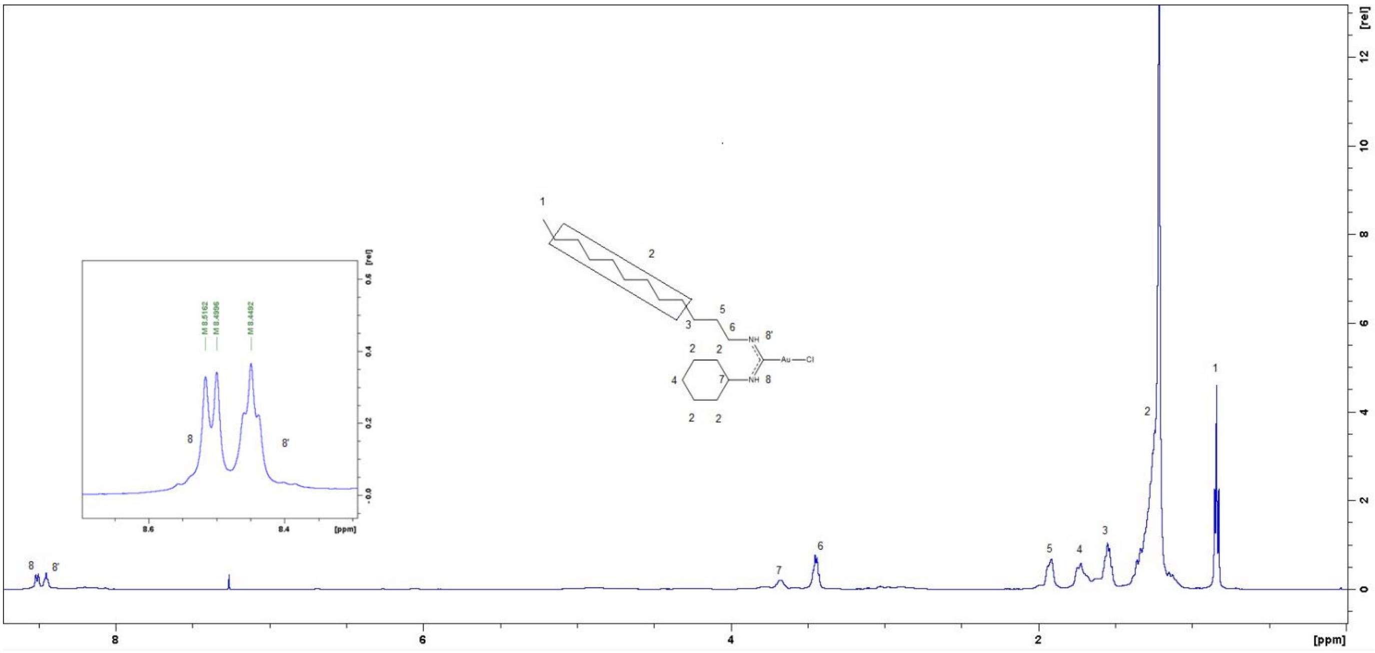

Fig. S 11. ^{1}H NMR spectrum of (*N'*-(cyclohexyl)-*N*-dodecylcarbamimidoyl) gold(I) chloride (**3d**)

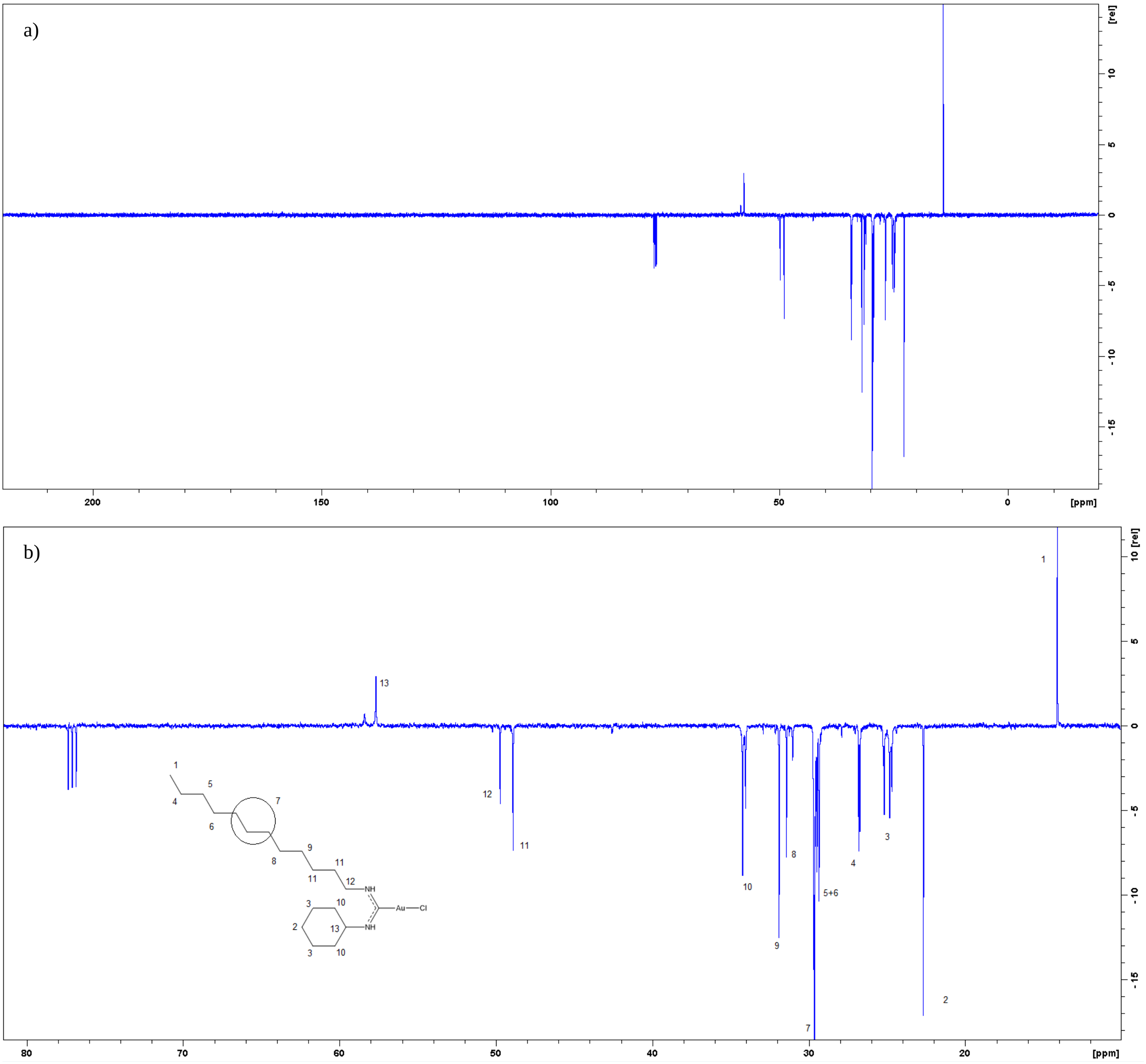

Fig. S 12. ^{13}C NMR spectrum of (*N'*-(cyclohexyl)-*N*-dodecylcarbamimidoyl) gold(I) chloride (**3d**). (a) Full overview of ^{13}C spectrum. (b) Expanded view of the 80 ppm-0 ppm region.

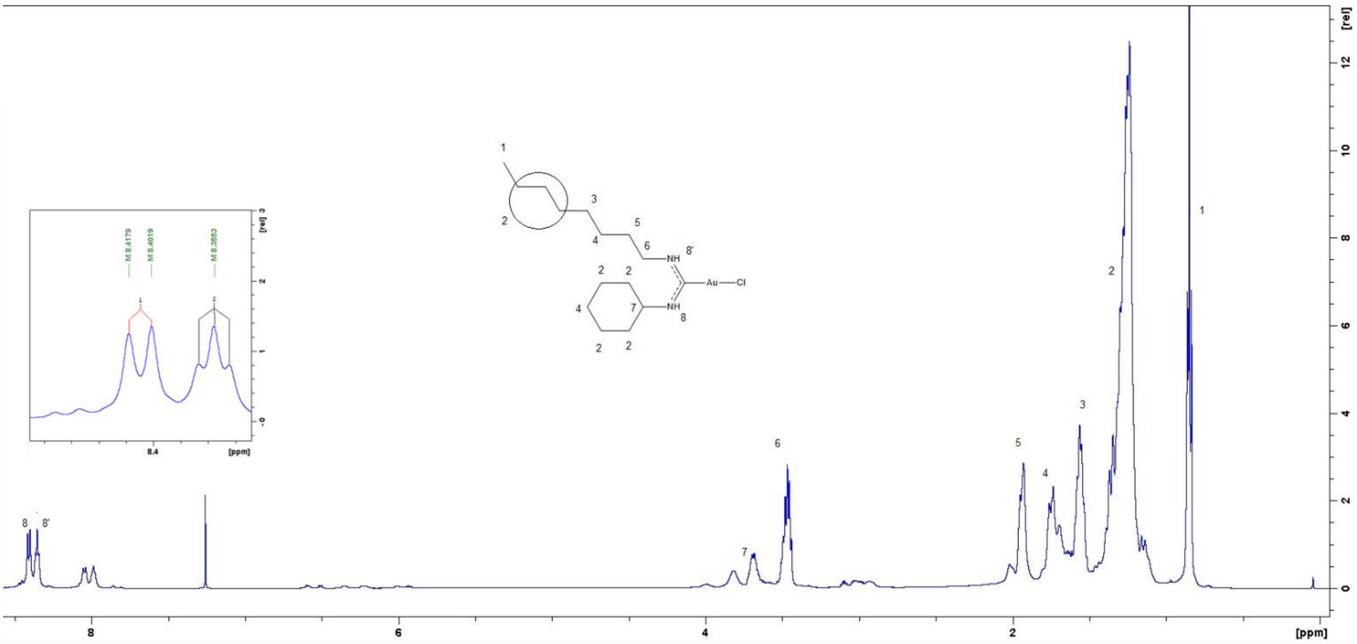

Fig. S 13. ^{1}H NMR spectrum of (*N’*-(cyclohexyl)-*N*-octylcarbamimidoyl) gold(I) chloride (**3e**)

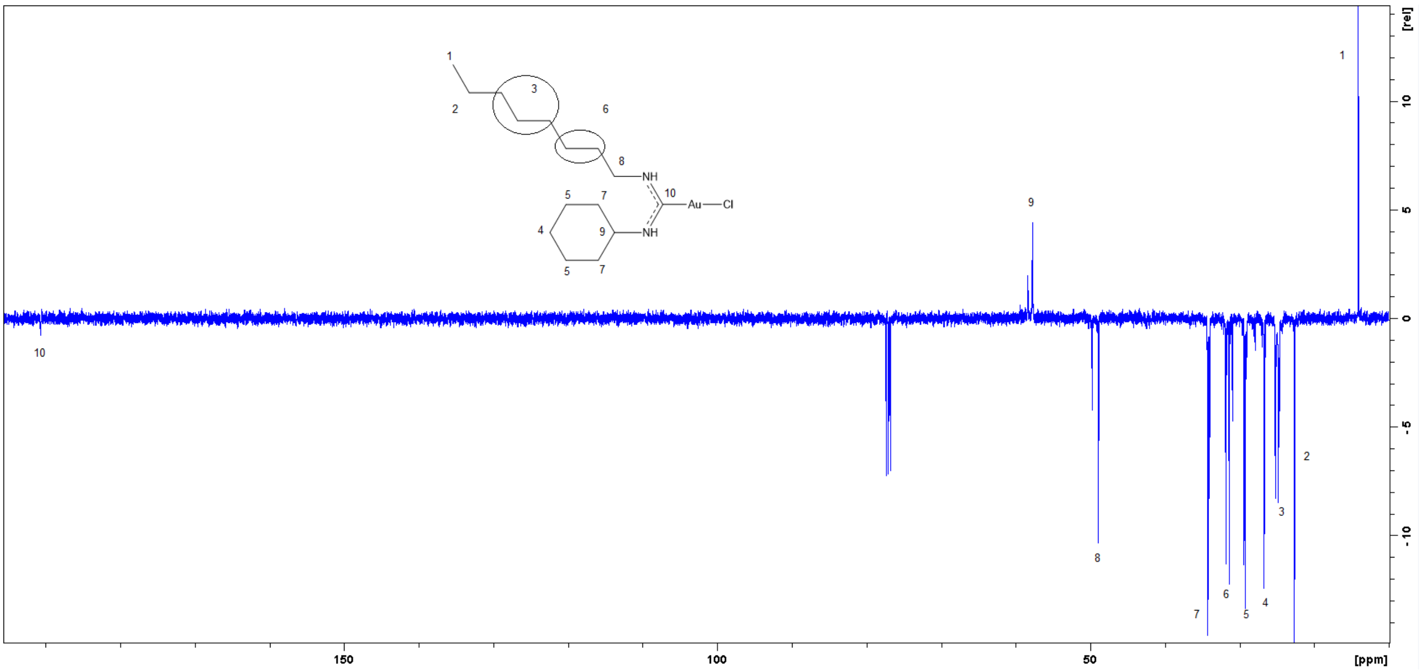

Fig. S 14. ^{13}C NMR spectrum of (*N'*-(cyclohexyl)-*N*-octylcarbamimidoyl) gold(I) chloride (**3e**)

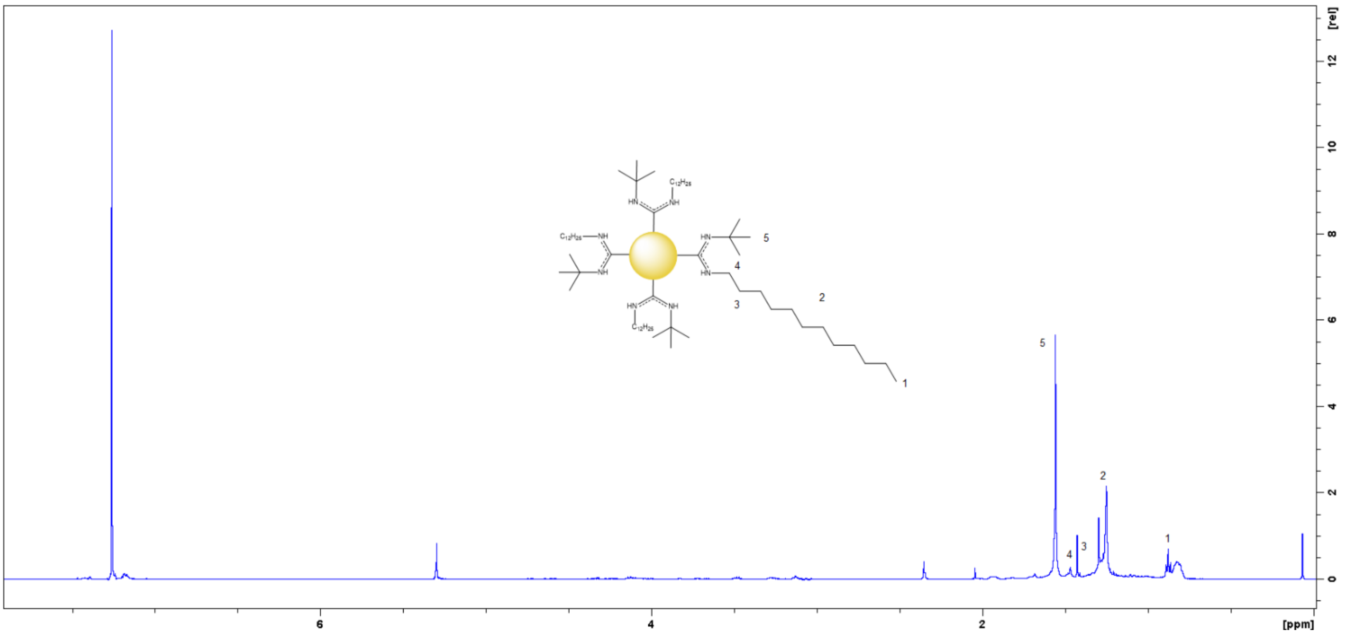

Fig. S 15. ^{1}H NMR spectrum of (*N'*-(^{t}butyl)-*N*-dodecylcarbamimidoyl) gold nanoparticles (**4a**)

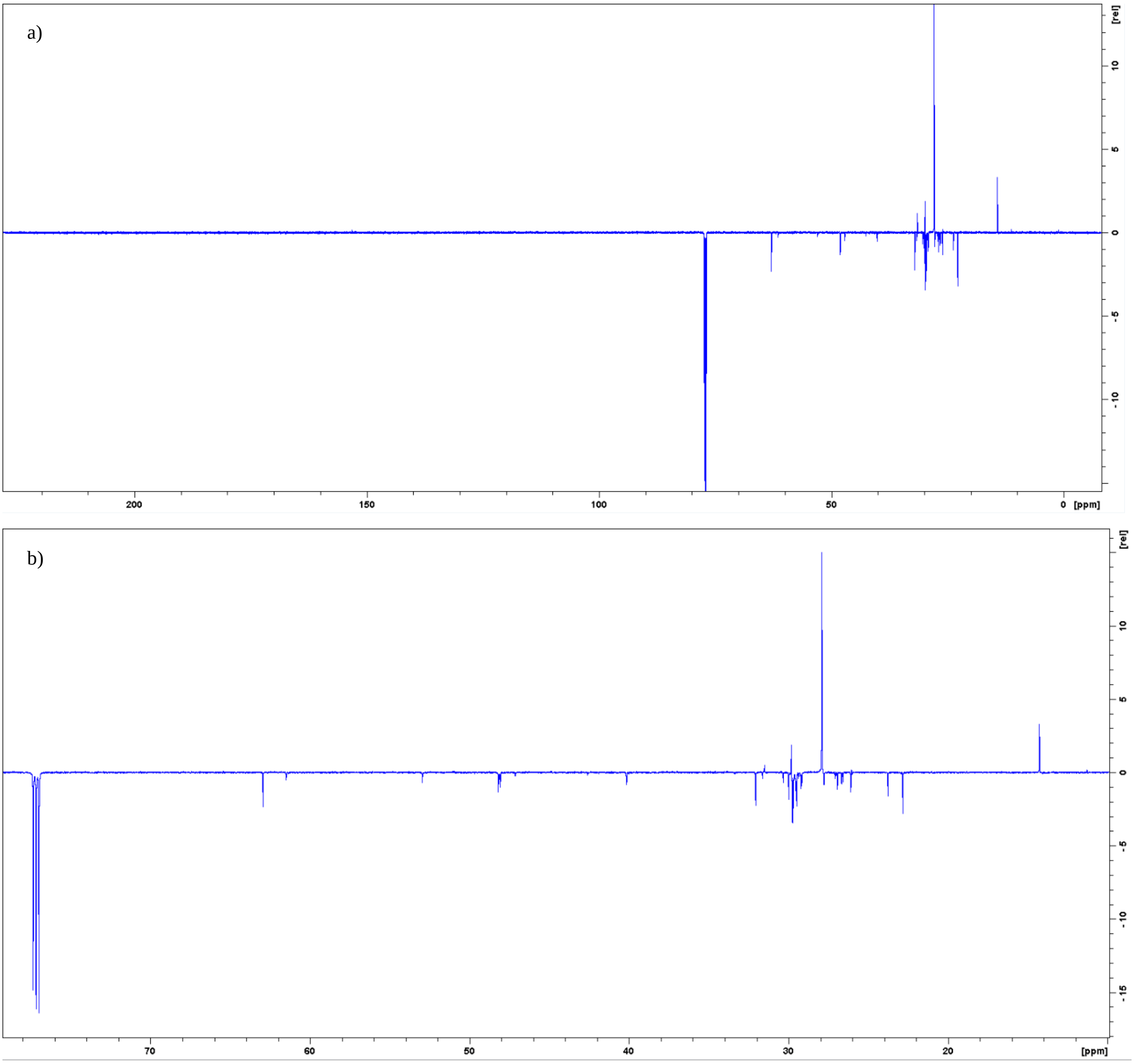

Fig. S 16. ^{13}C NMR spectrum of (*N'*-(^{t}butyl)-*N*-dodecylcarbamimidoyl) gold nanoparticles (**4a**). (a) Full overview of ^{13}C spectrum. (b) Expanded view of the 80 ppm-0 ppm region.

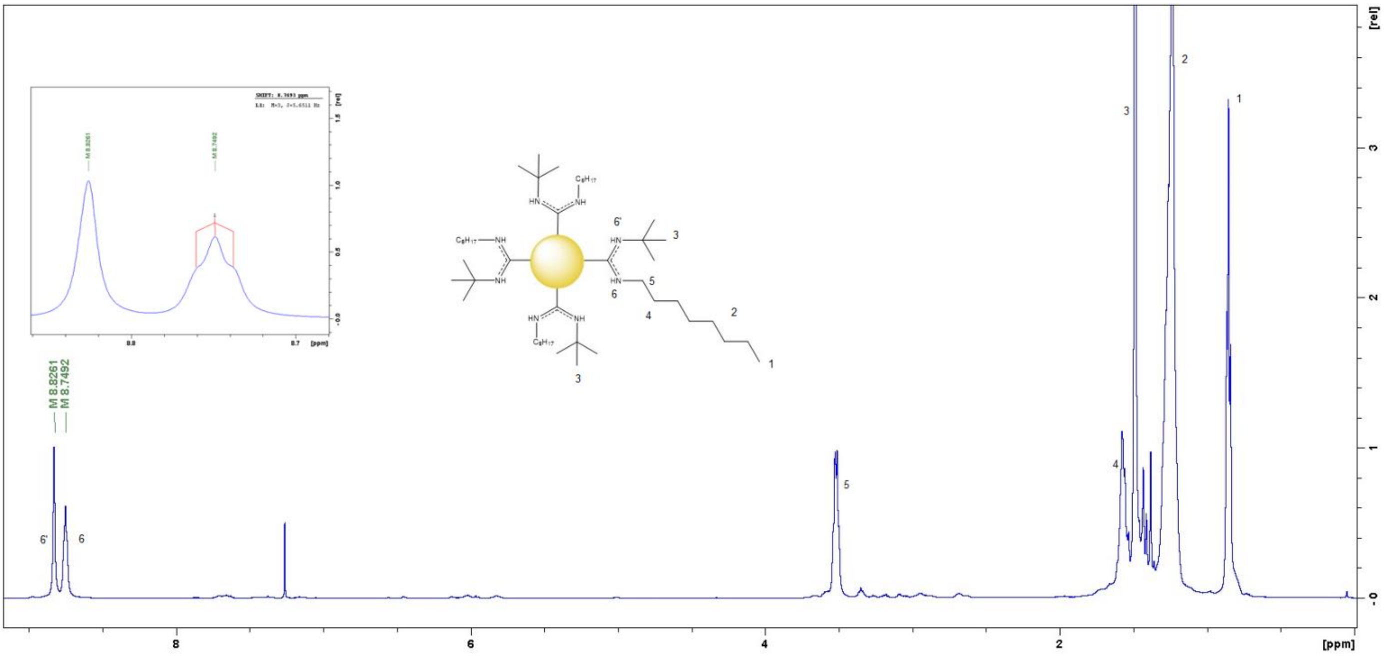

Fig. S 17. ^{1}H NMR spectrum of (*N'*-(^{t}butyl)-*N*-octylcarbamimidoyl) gold nanoparticles (**4b**)

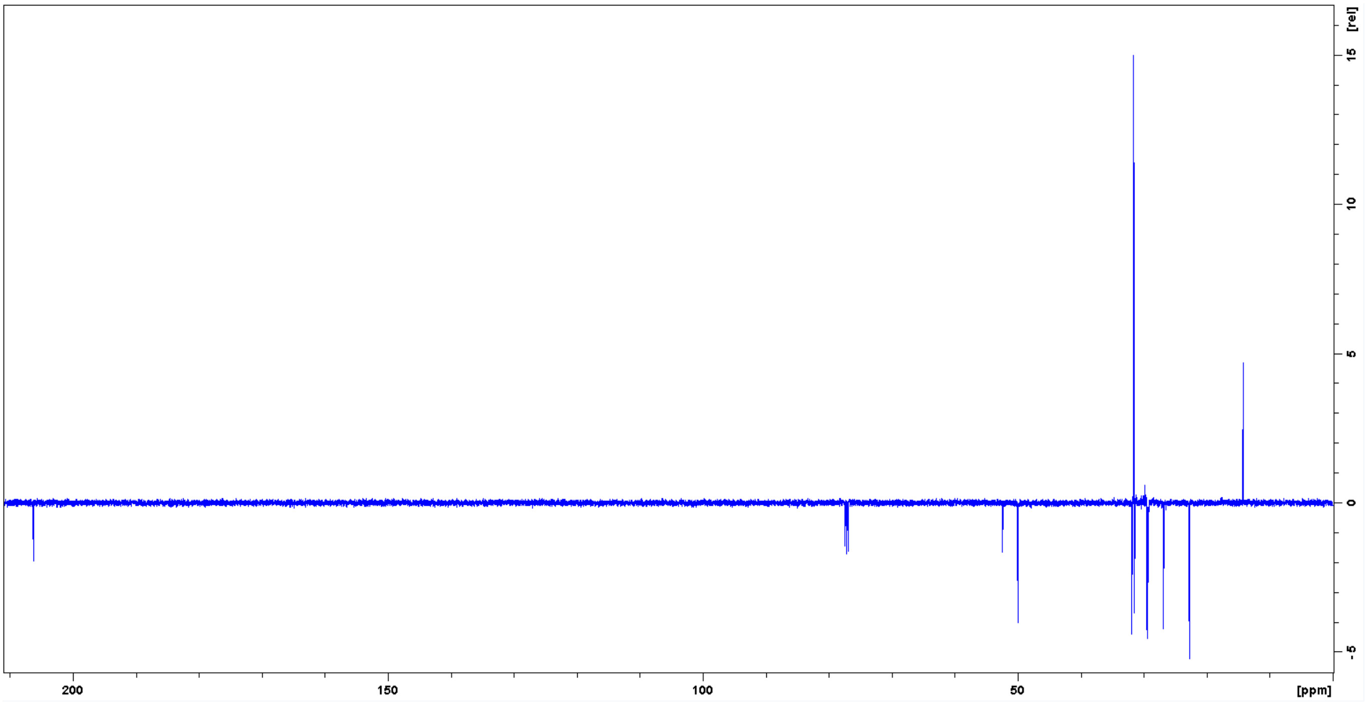

Fig. S 18. ^{13}C NMR spectrum of (*N'*-(^{t}butyl)-*N*-octylcarbamimidoyl) gold nanoparticles (**4b**)

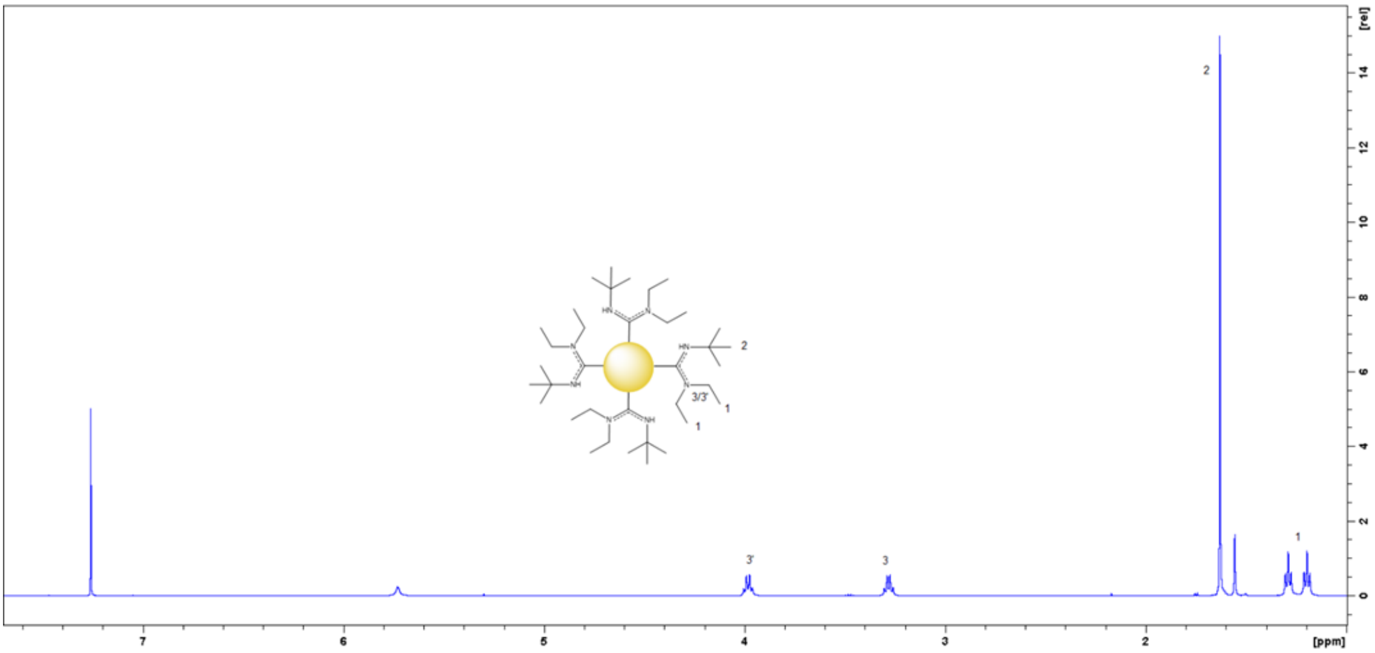

Fig. S 19. ^{1}H NMR spectrum of (*N*'-(^{t}butyl)-*N*,*N*-diethylcarbamimidoyl) gold nanoparticles (**4c**)

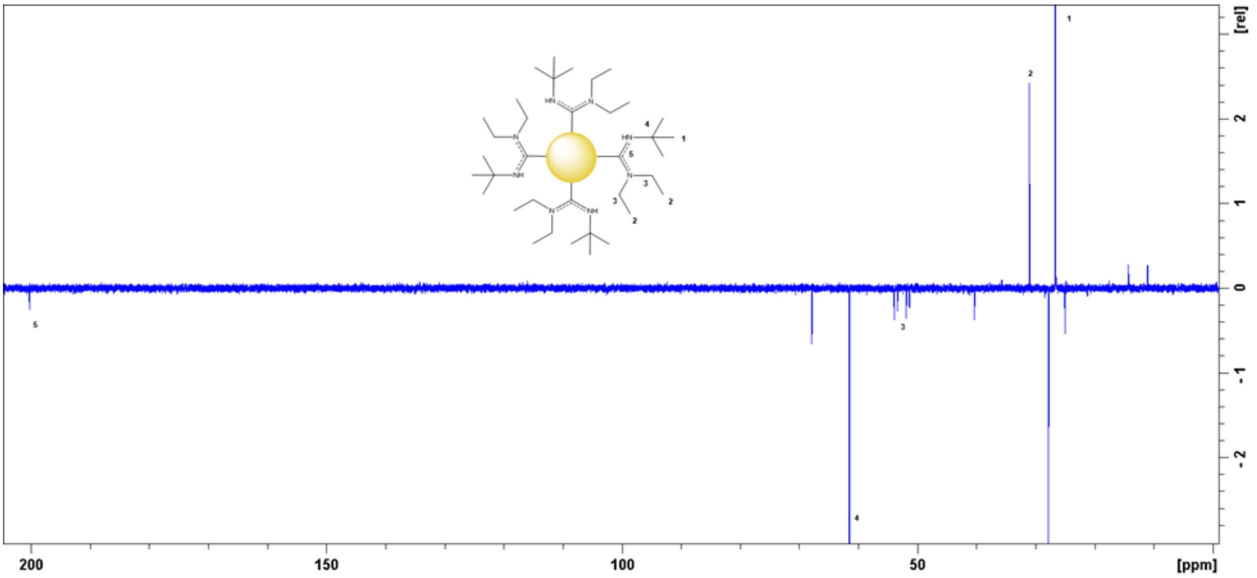

Fig. S 20. ^{13}C NMR spectrum of (*N*'-(^{t}butyl)-*N*,*N*-diethylcarbamimidoyl) gold nanoparticles (**4c**)

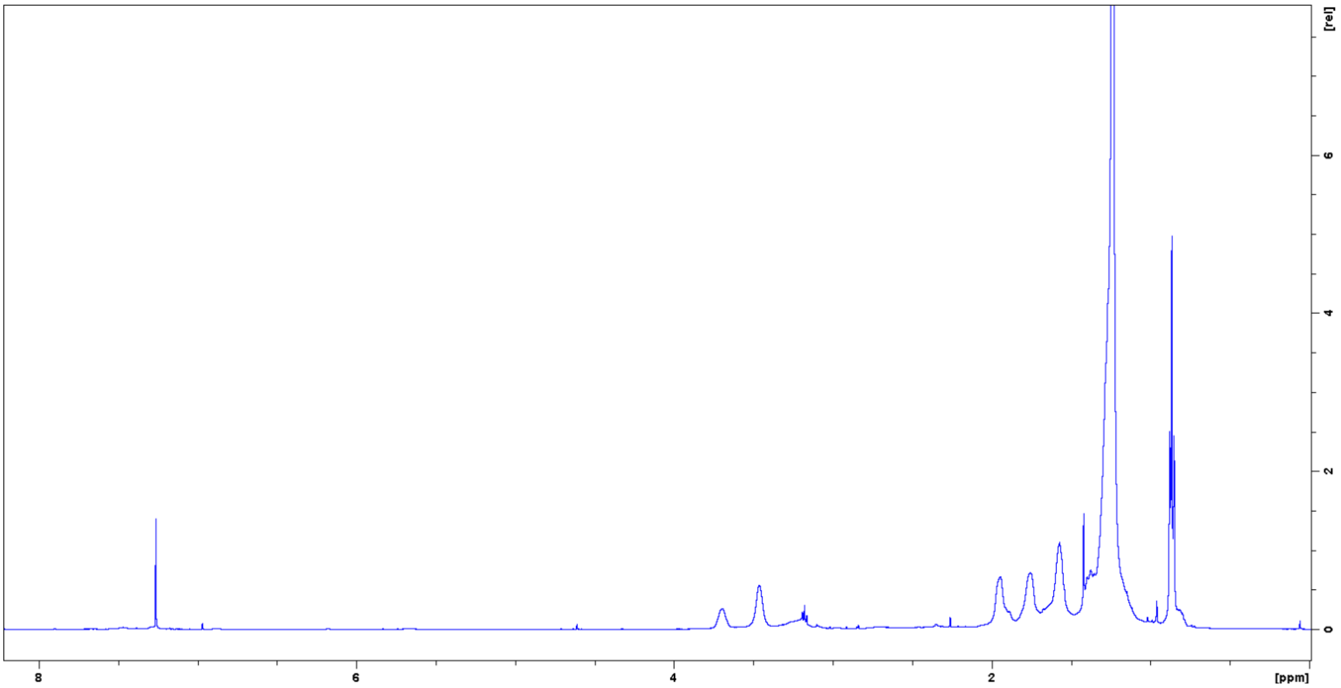

Fig. S 21. ^{1}H NMR spectrum of (*N'*-(cyclohexyl)-*N*-dodecylcarbamimidoyl) gold nanoparticles (**4d**)

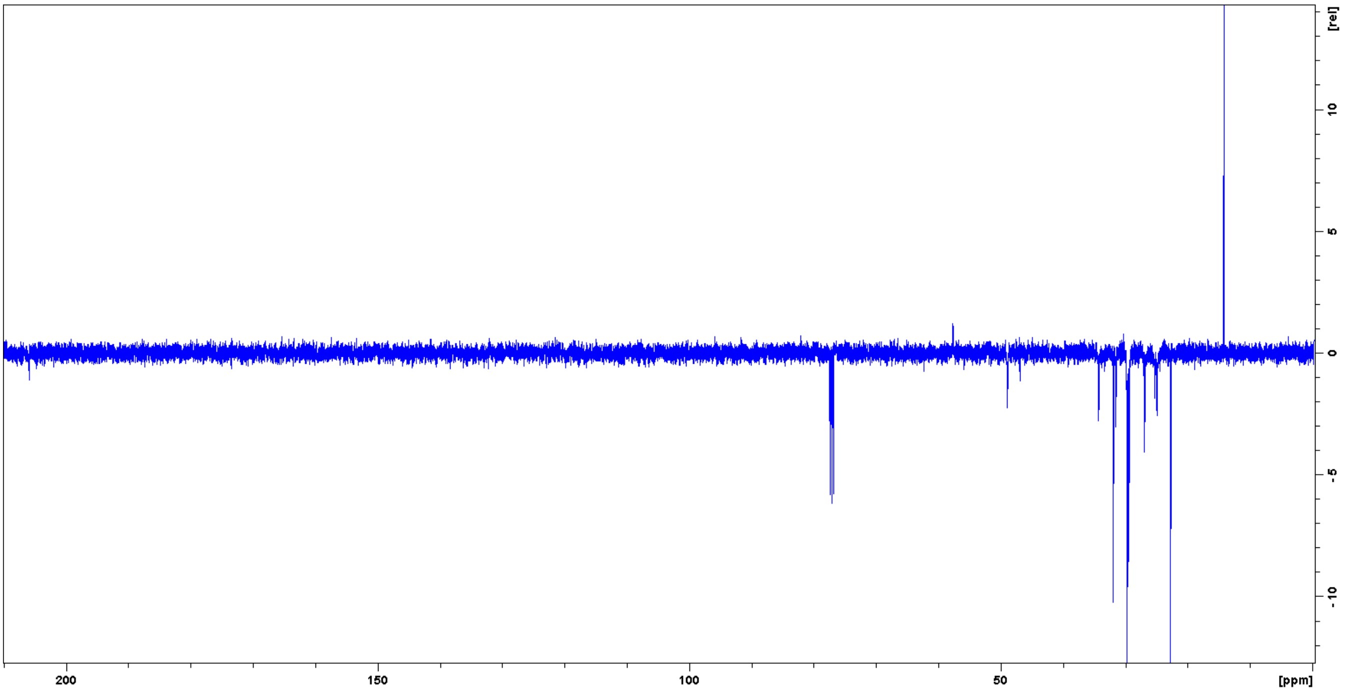

Fig. S 22. ^{13}C NMR spectrum of (*N'*-(cyclohexyl)-*N*-dodecylcarbamimidoyl) gold nanoparticles (**4d**)

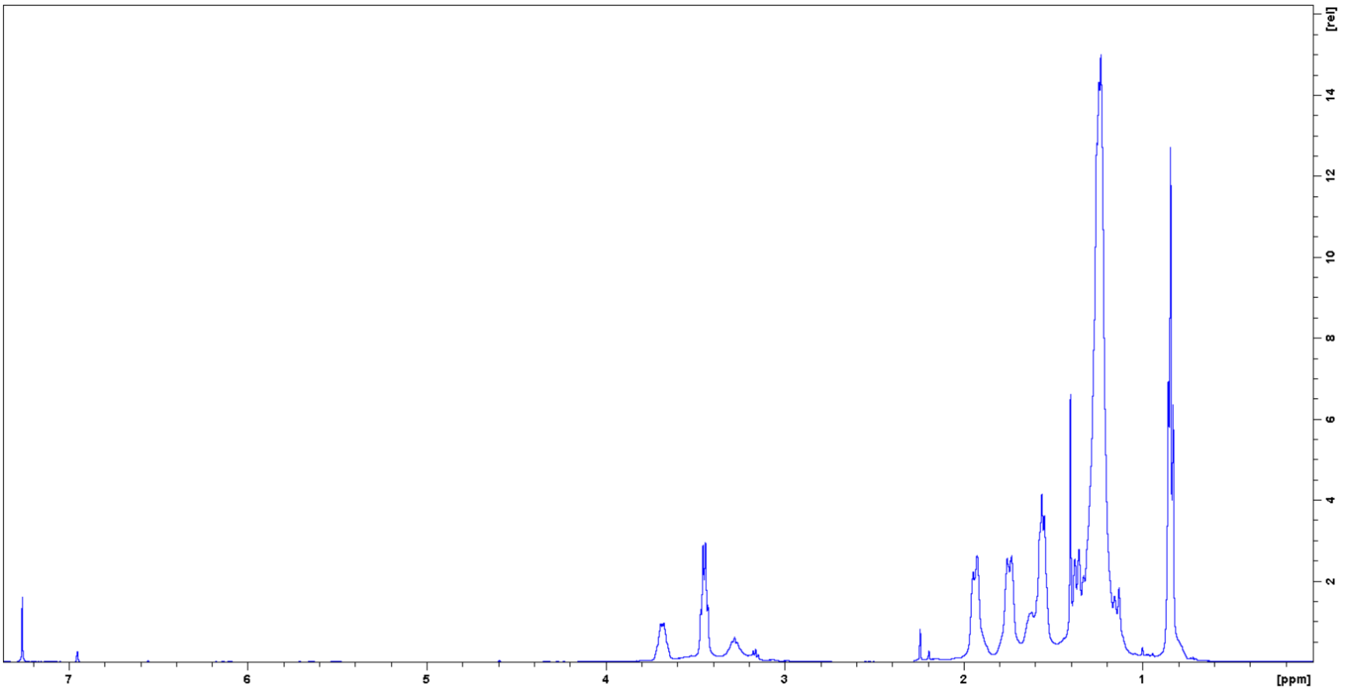

Fig. S 23. ^{1}H NMR spectrum of (*N'*-(cyclohexyl)-*N*-octylcarbamimidoyl) gold nanoparticles (**4e**)

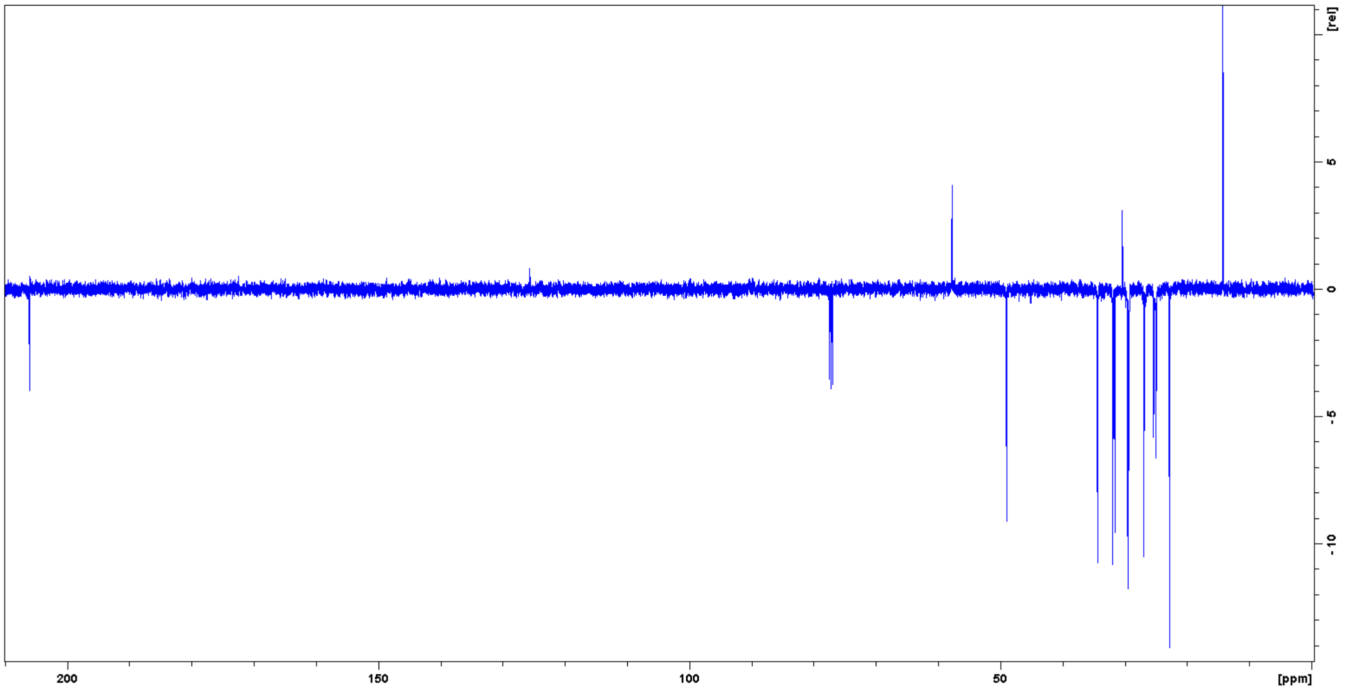

Fig. S 24. ^{13}C NMR spectrum of (*N'*-(cyclohexyl)-*N*-octylcarbamimidoyl) gold nanoparticles (**4e**)

UV-Vis Stability Studies

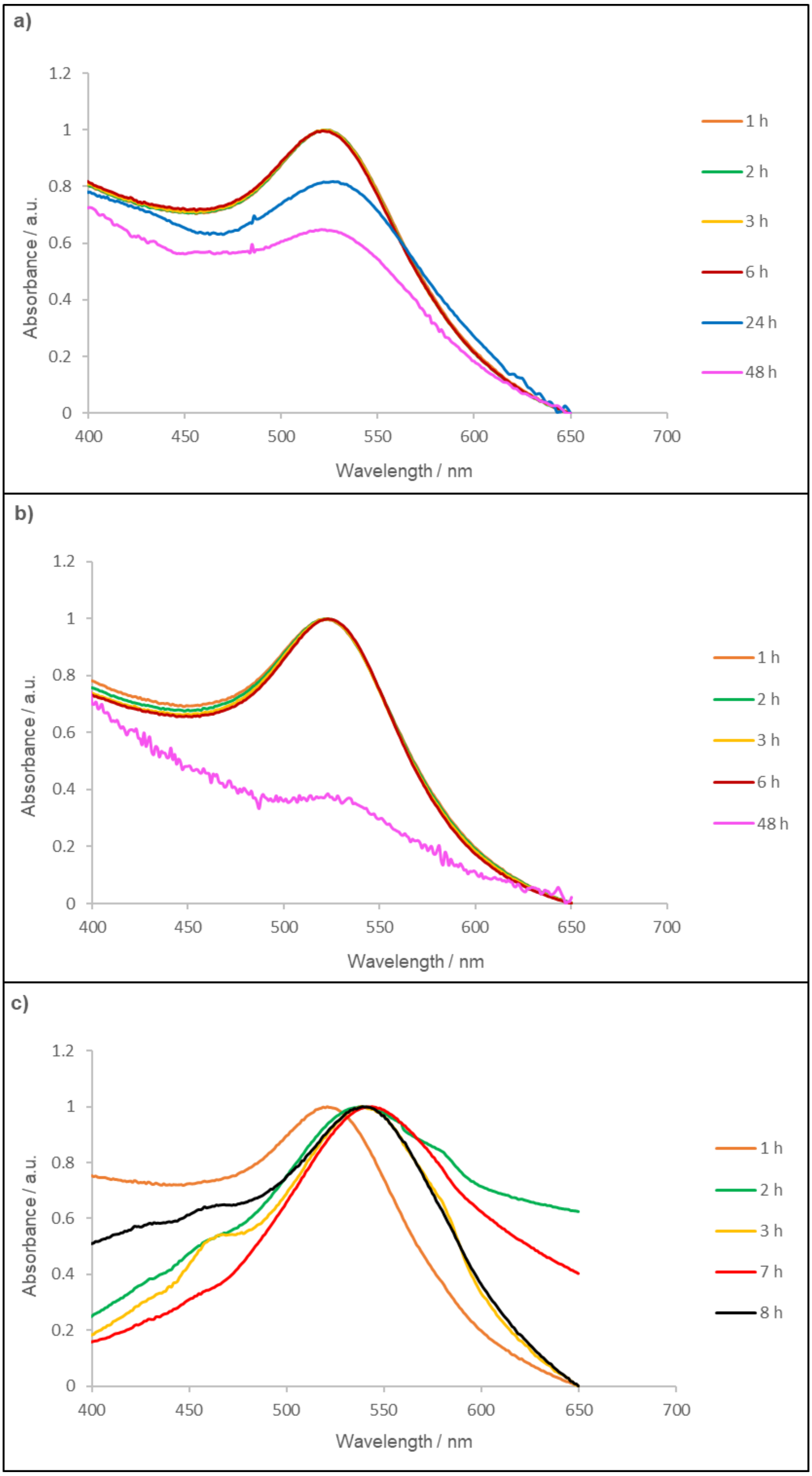

Fig. S 25. UV-Vis absorption spectra of compound **4b** in toluene. a) 25 °C; b) 50 °C; c) 80 °C.

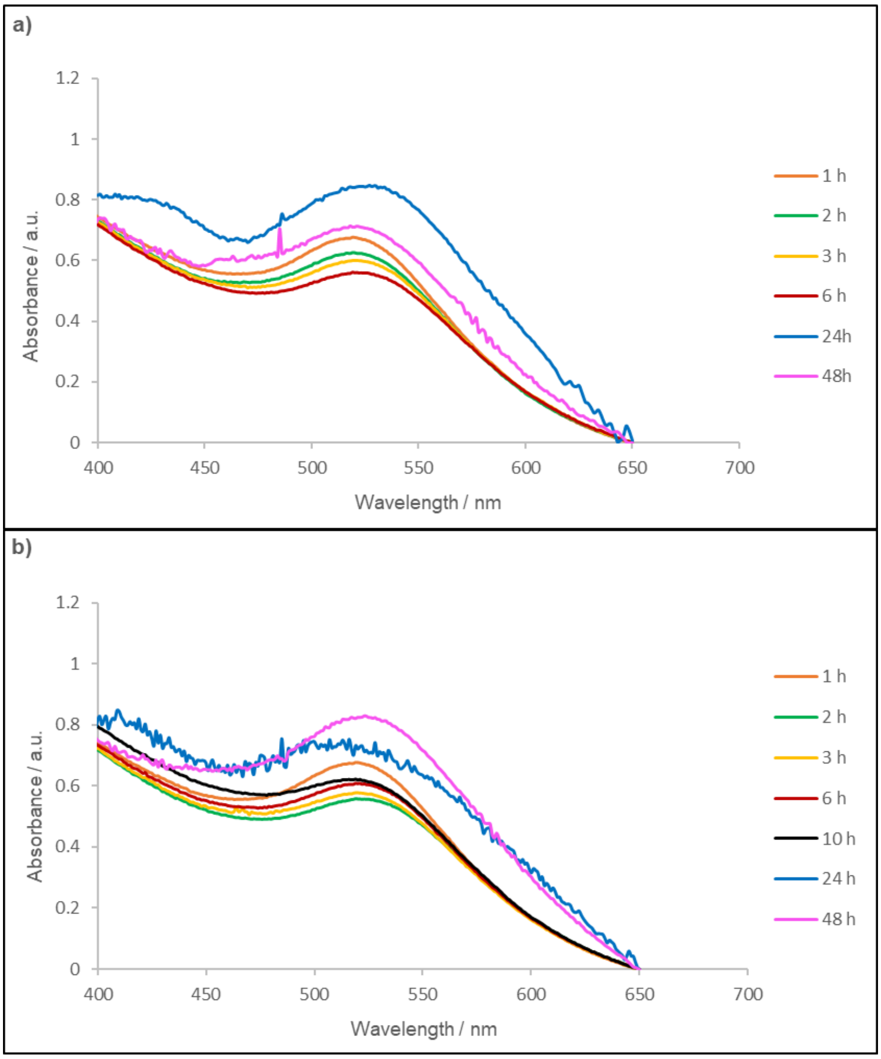

Fig. S 26. UV-Vis absorption spectra of compound **4b** in a 10 mM 1-dodecanethiol toluene solution. a) 25 °C; b) 50 °C .

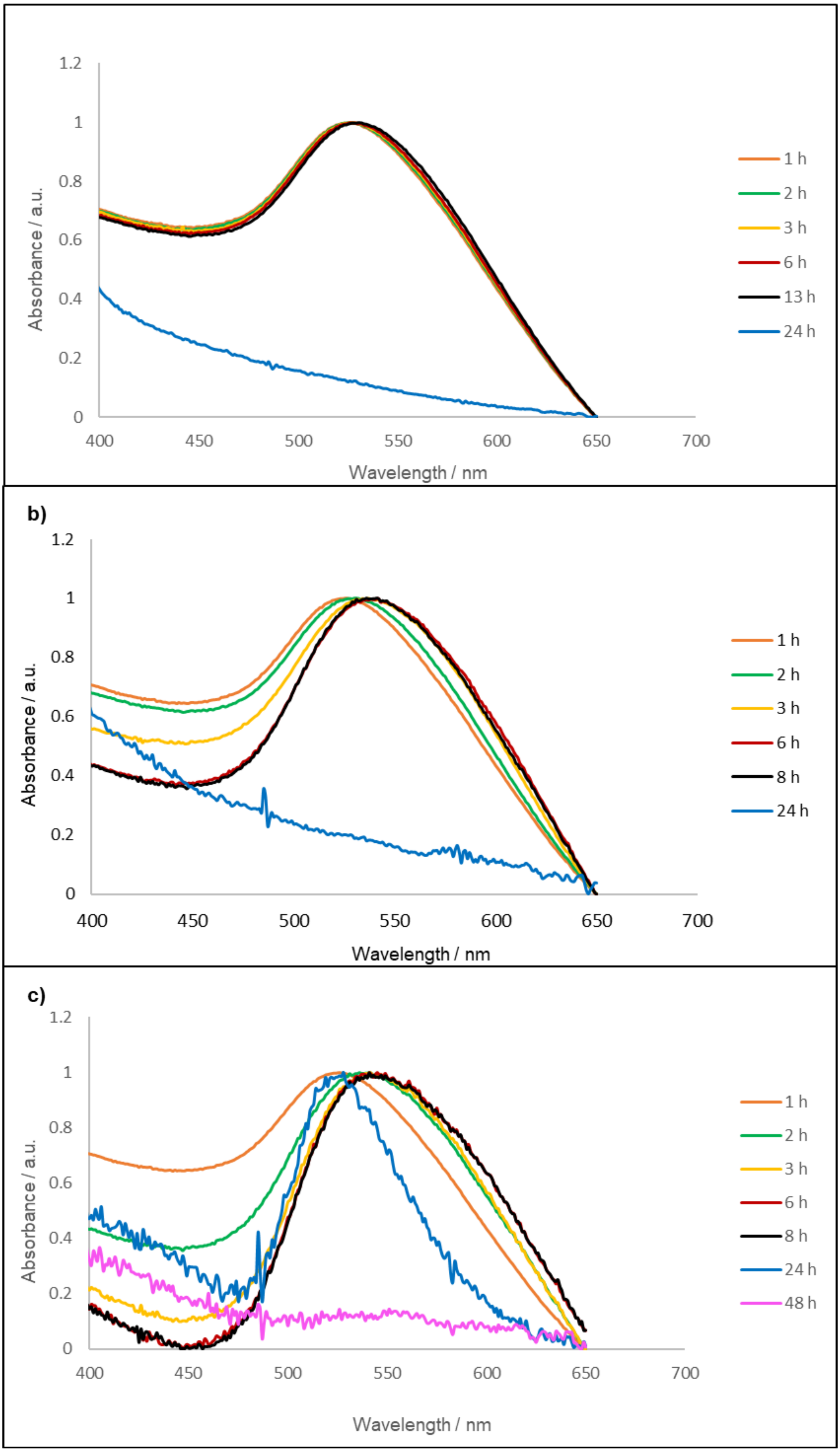

Fig. S 27. UV-Vis absorption spectra of compound **4c** in water. a) 25 °C; b) 50 °C; c) 80 °C.

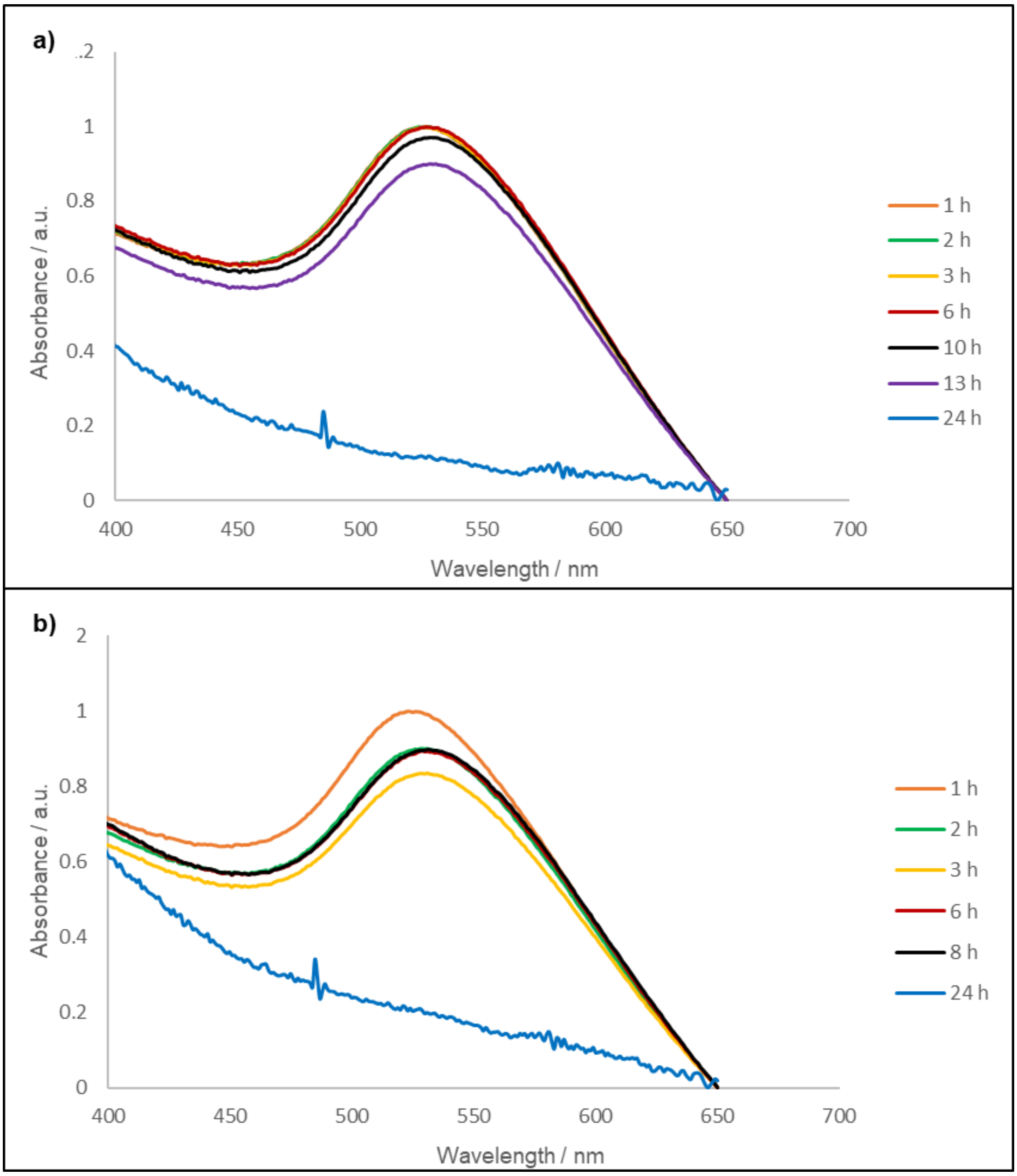

Fig. S 28. UV-Vis absorption spectra of compound **4c** in a 10 mM GSH water solution at pH 7.4. a) 25 °C; b) 50 °C .

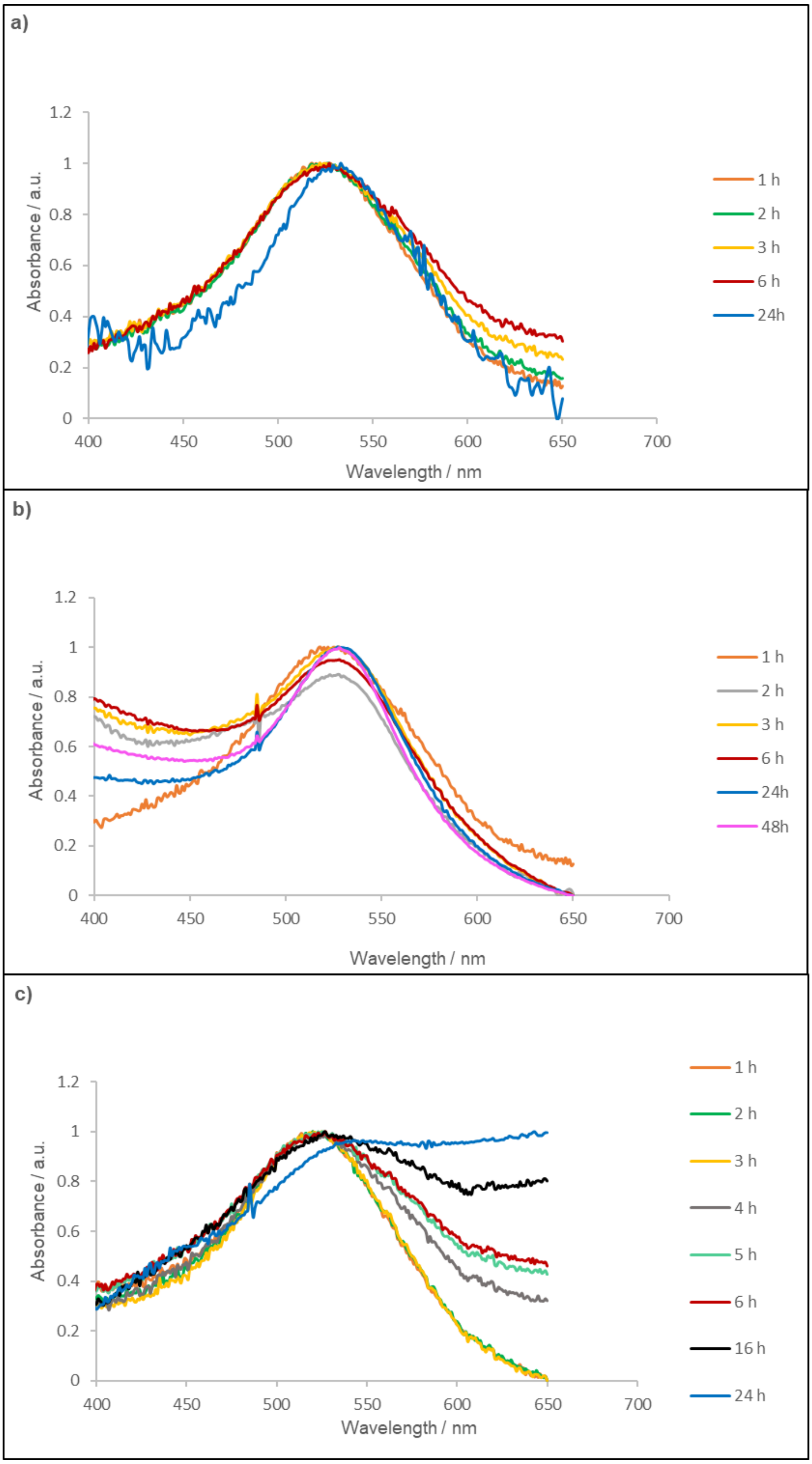

Fig. S 29. UV-Vis absorption spectra of compound **4d** in toluene. a) 25 °C; b) 50 °C; c) 80 °C.

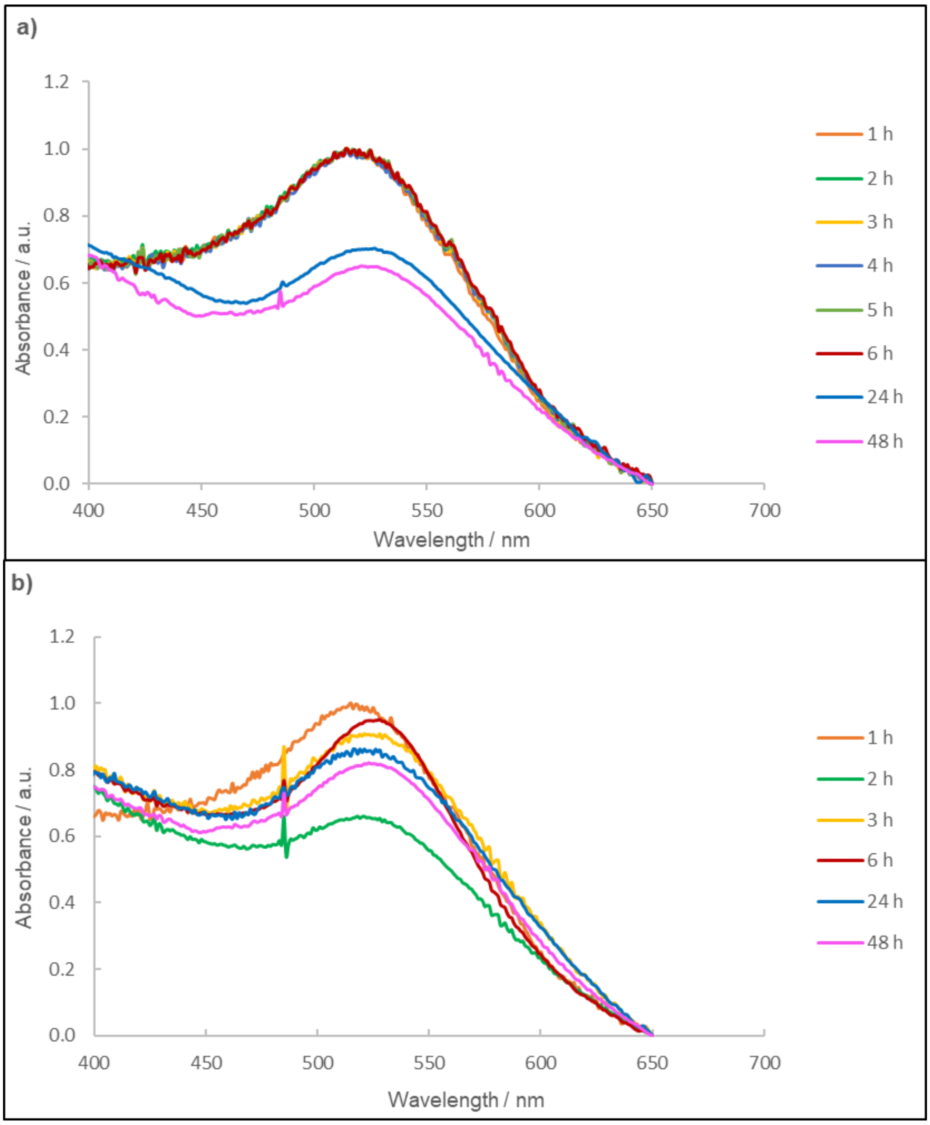

Fig. S 30. UV-Vis absorption spectra of compound **4d** in a 10 mM 1-dodecanethiol toluene solution. a) 25 °C; b) 50 °C.

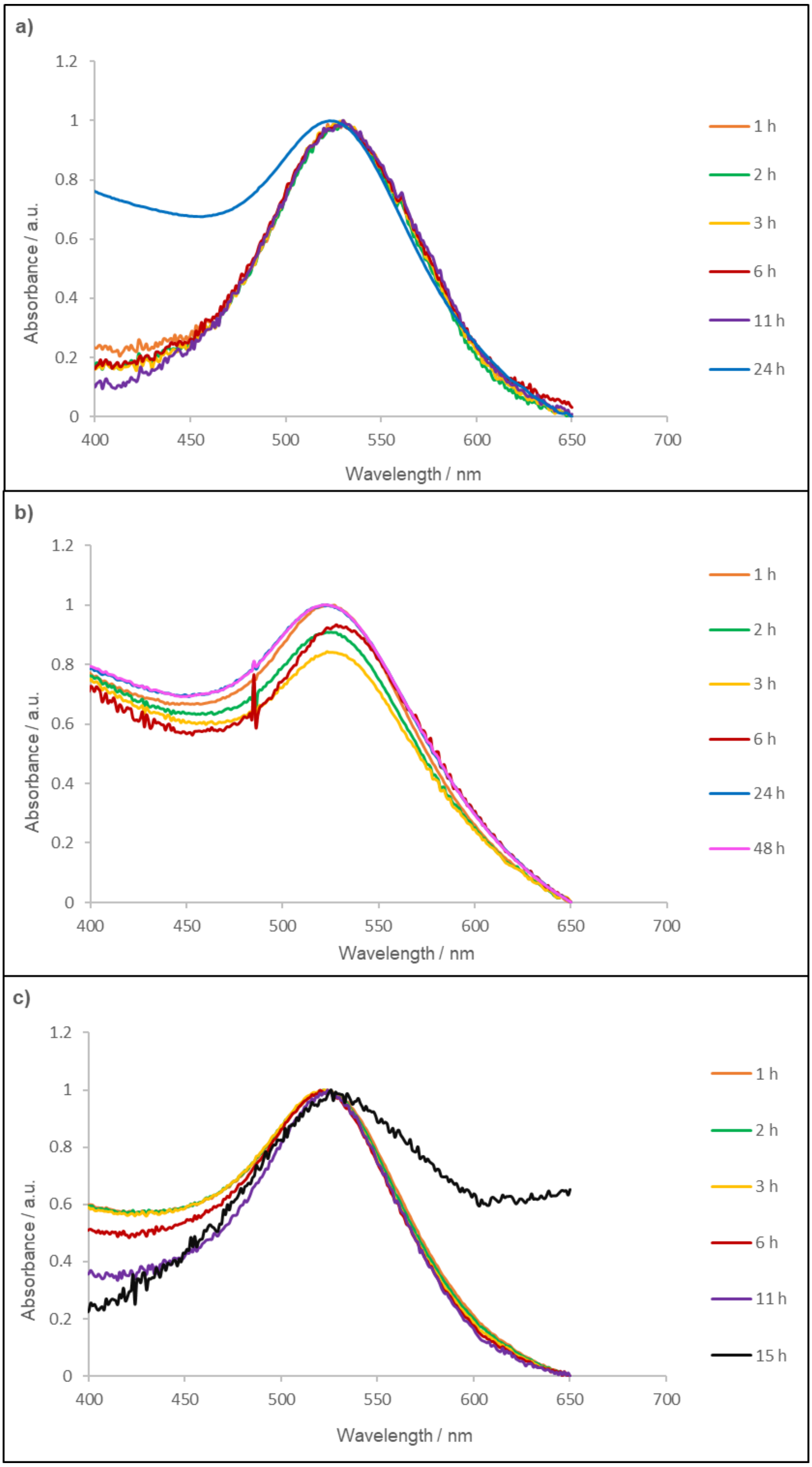

Fig. S 31. UV-Vis absorption spectra of compound **4e** in toluene. a) 25 °C; b) 50 °C; c) 80 °C.

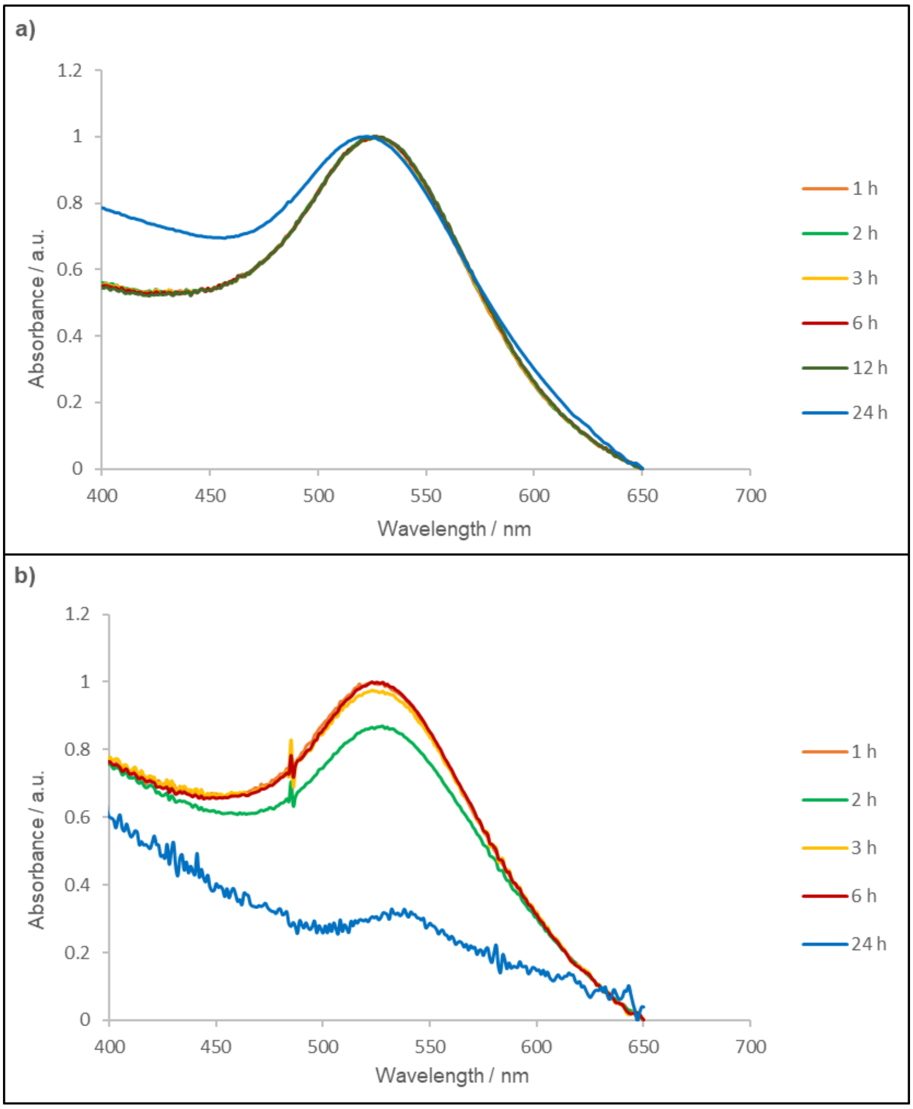

Fig. S 32. UV-Vis absorption spectra of compound **4e** in a 10 mM 1-dodecanethiol toluene solution. a) 25 °C; b) 50 °C.

Thermo-gravimetric analysis

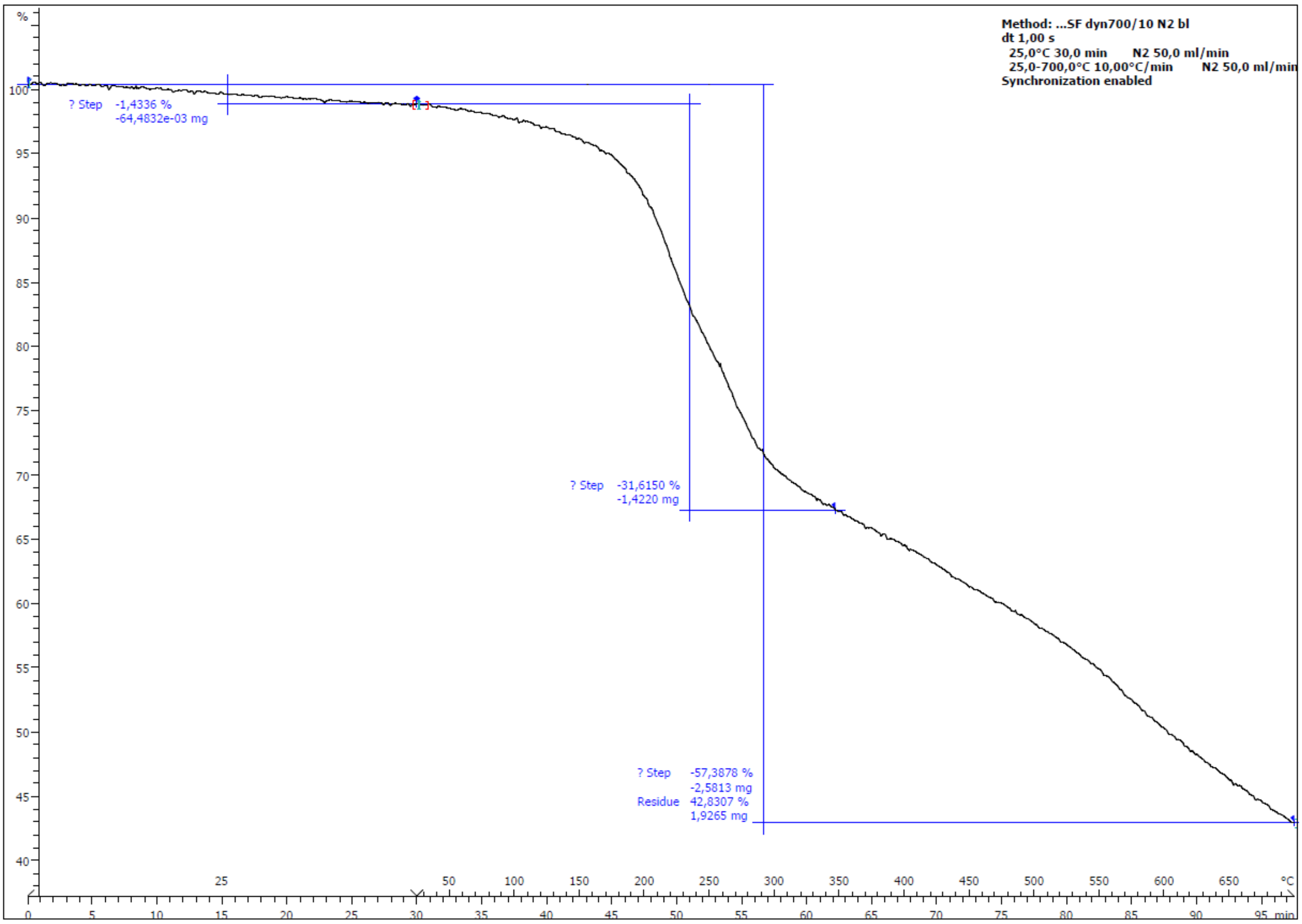

Fig. S 33. Thermogravimetric analysis of compound **4a**.

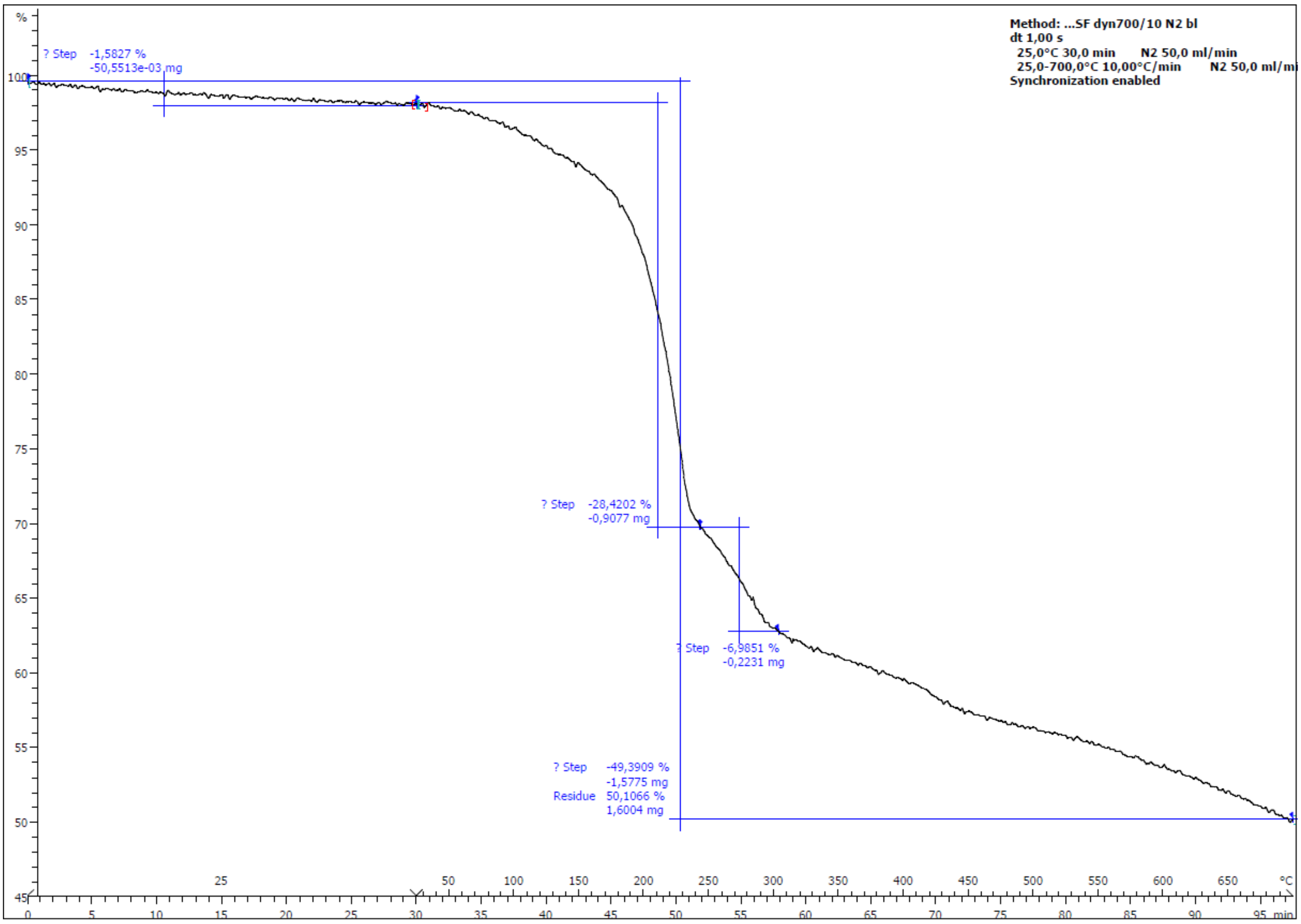

Fig. S 34. Thermogravimetric analysis of compound **4b.**

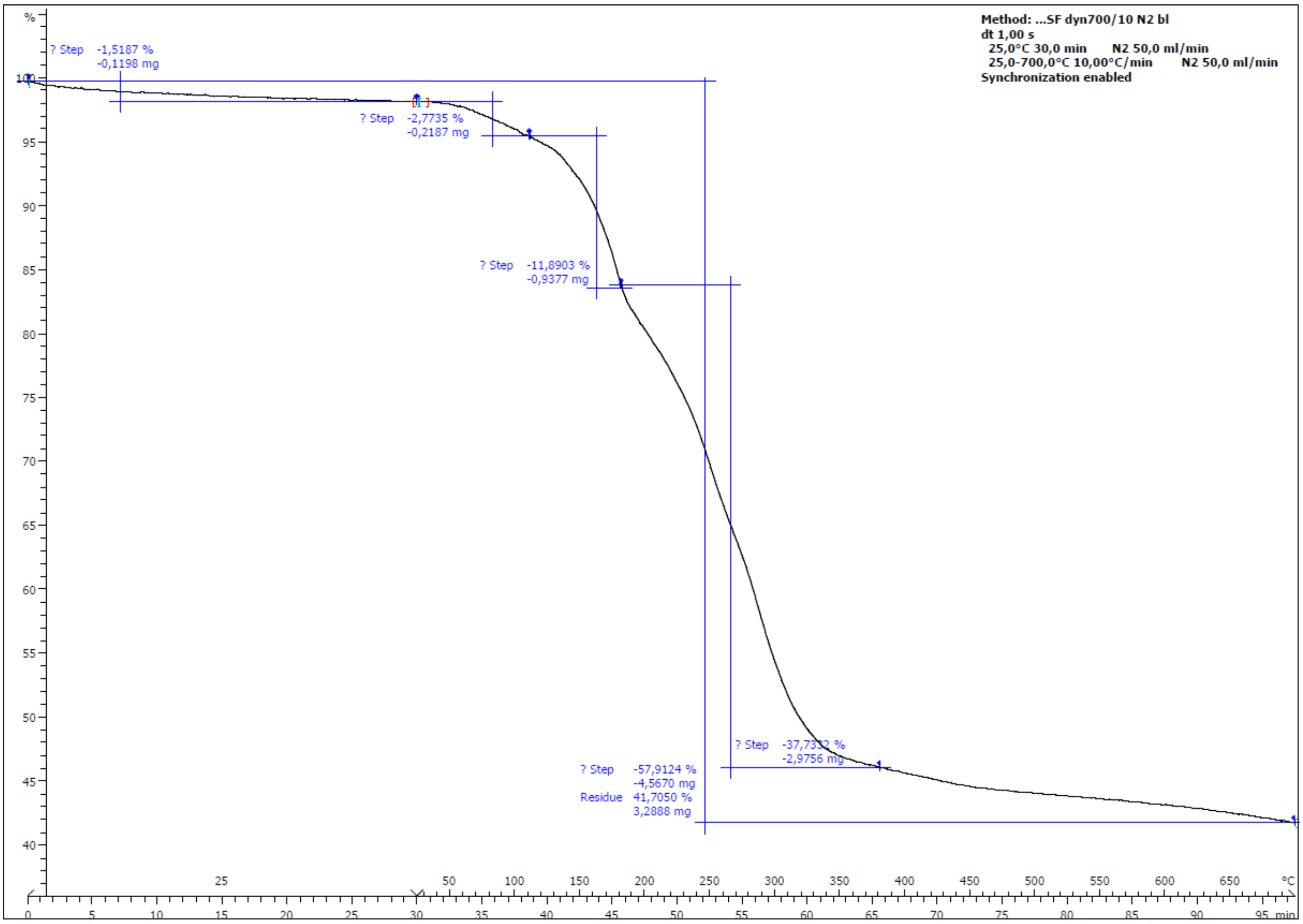

Fig. S 35. Thermogravimetric analysis of compound **4c.**

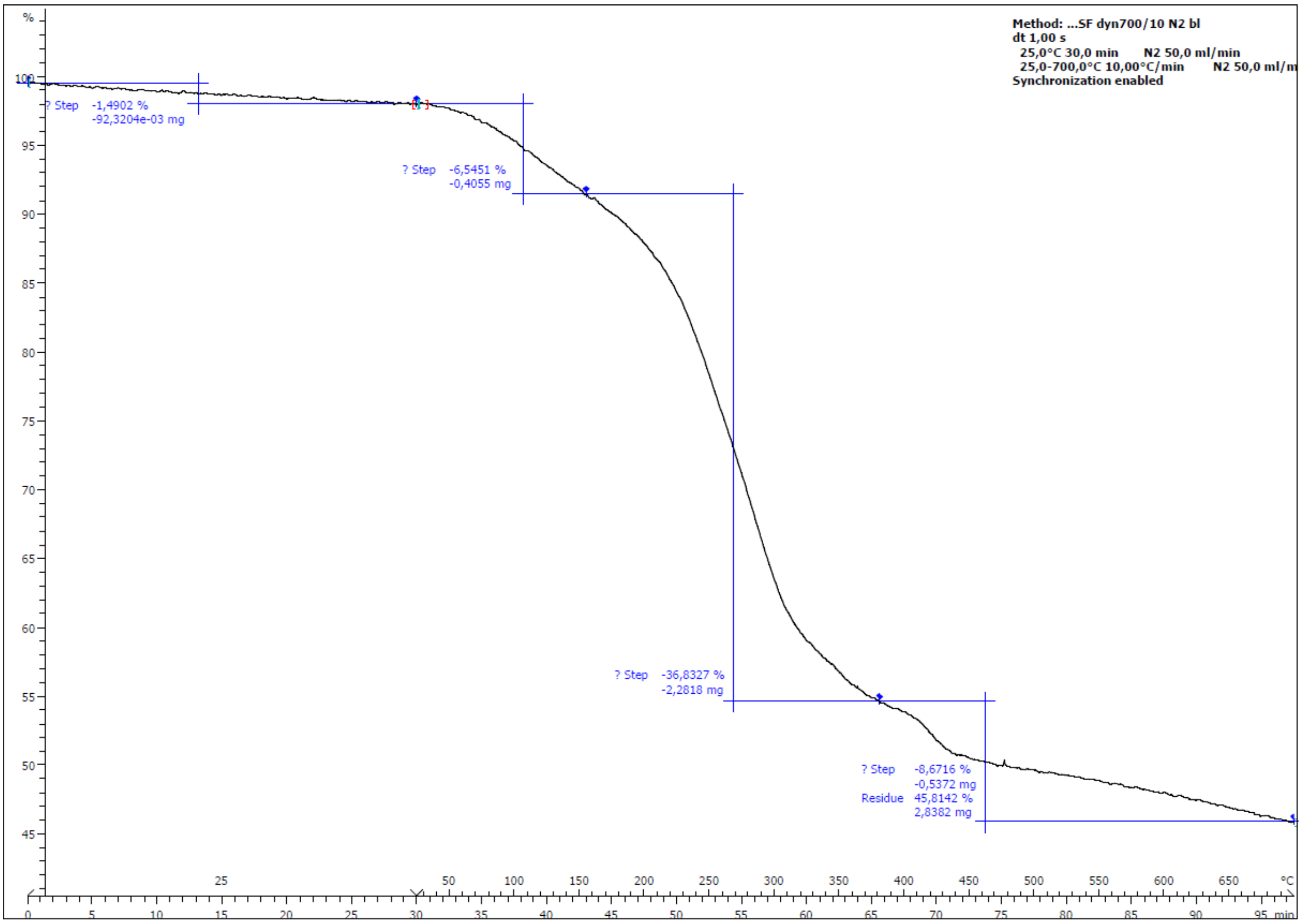

Fig. S 36. Thermogravimetric analysis of compound **4d**.

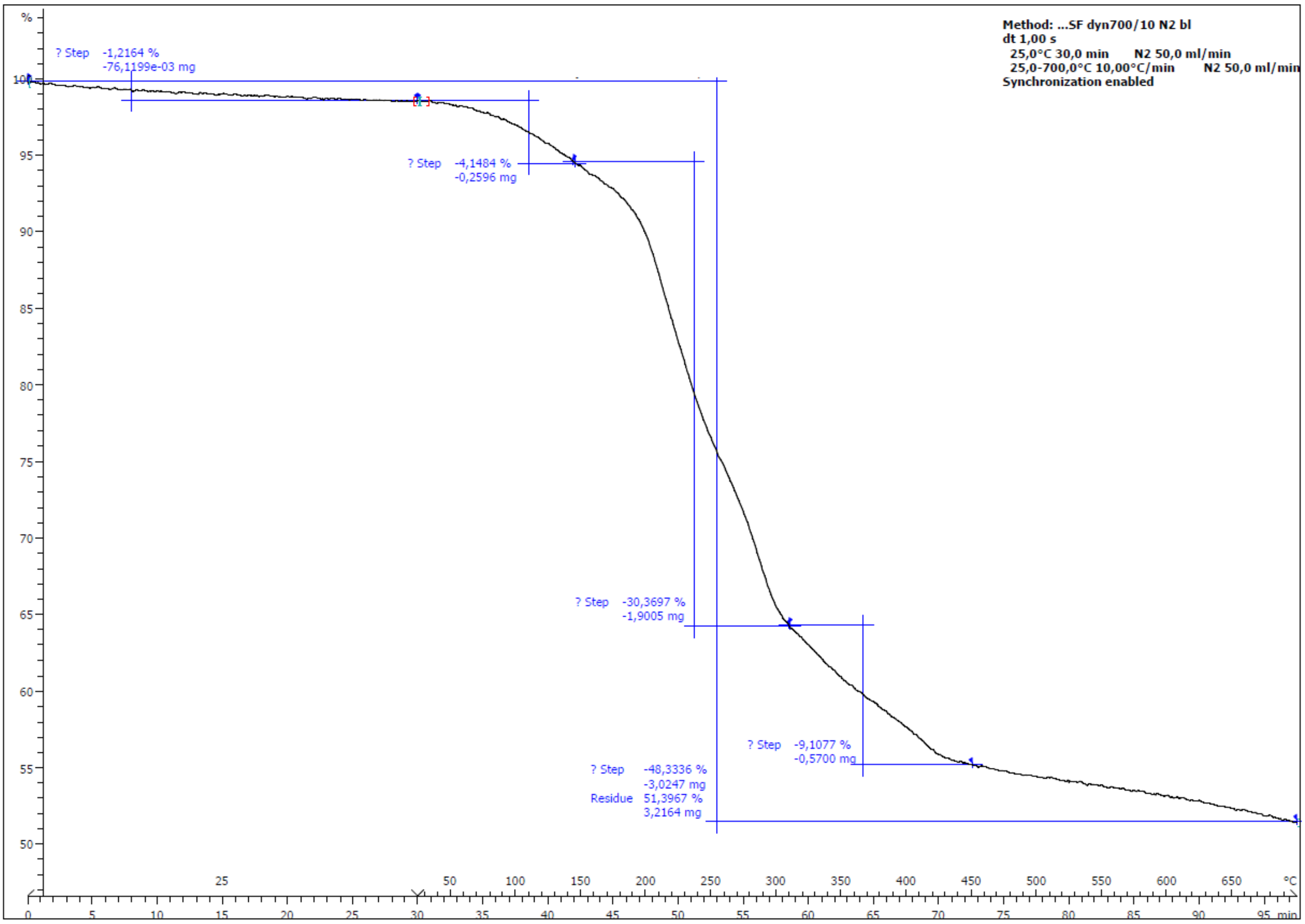

Fig. S 37. Thermogravimetric analysis of compound **4e**.

TEM micrographs

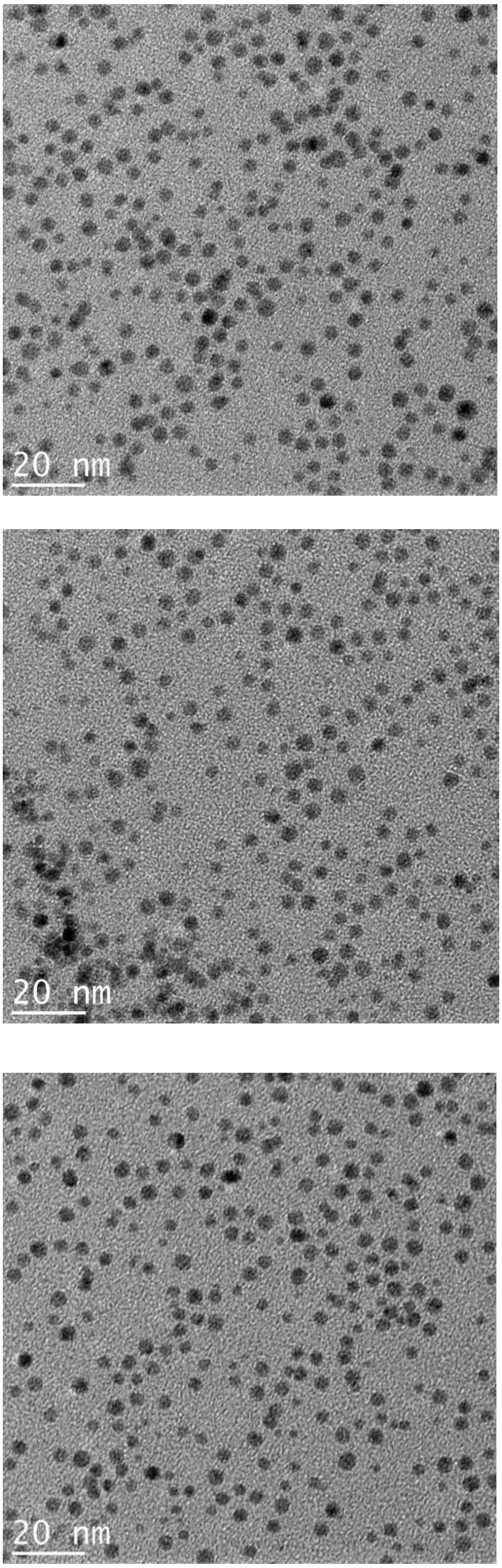

Fig. S 38. TEM images of **4a**.

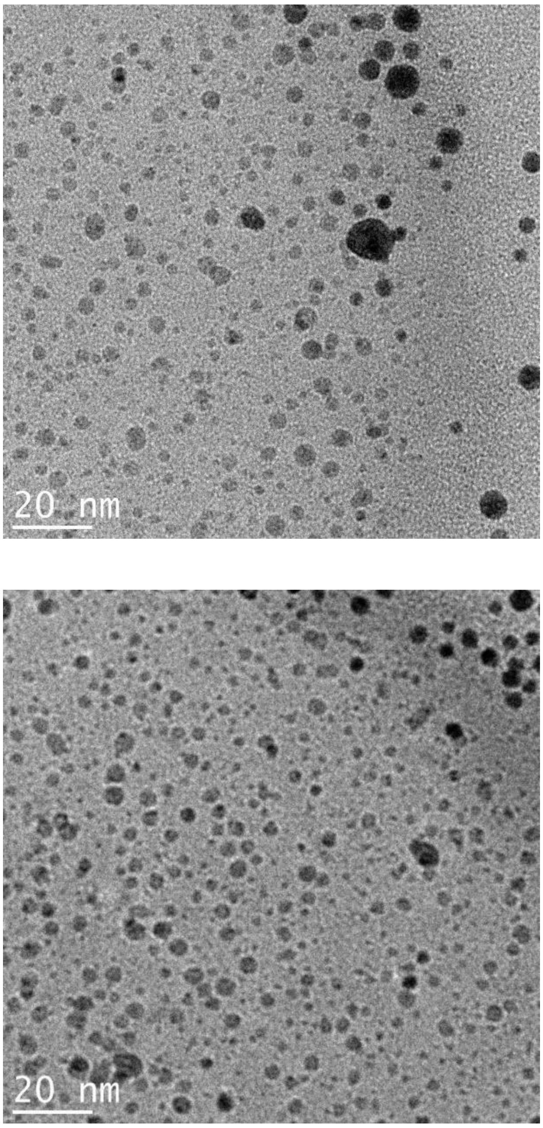

Fig. S 39. TEM images of **4b**.

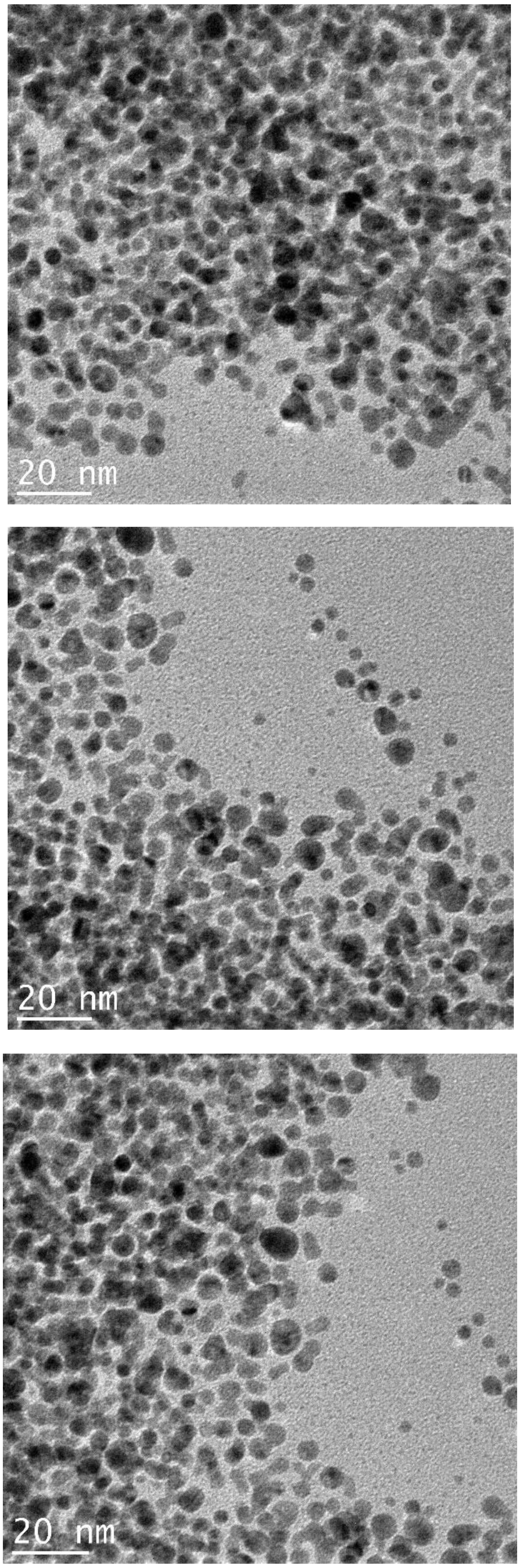

Fig. S 40. TEM images of **4c**.

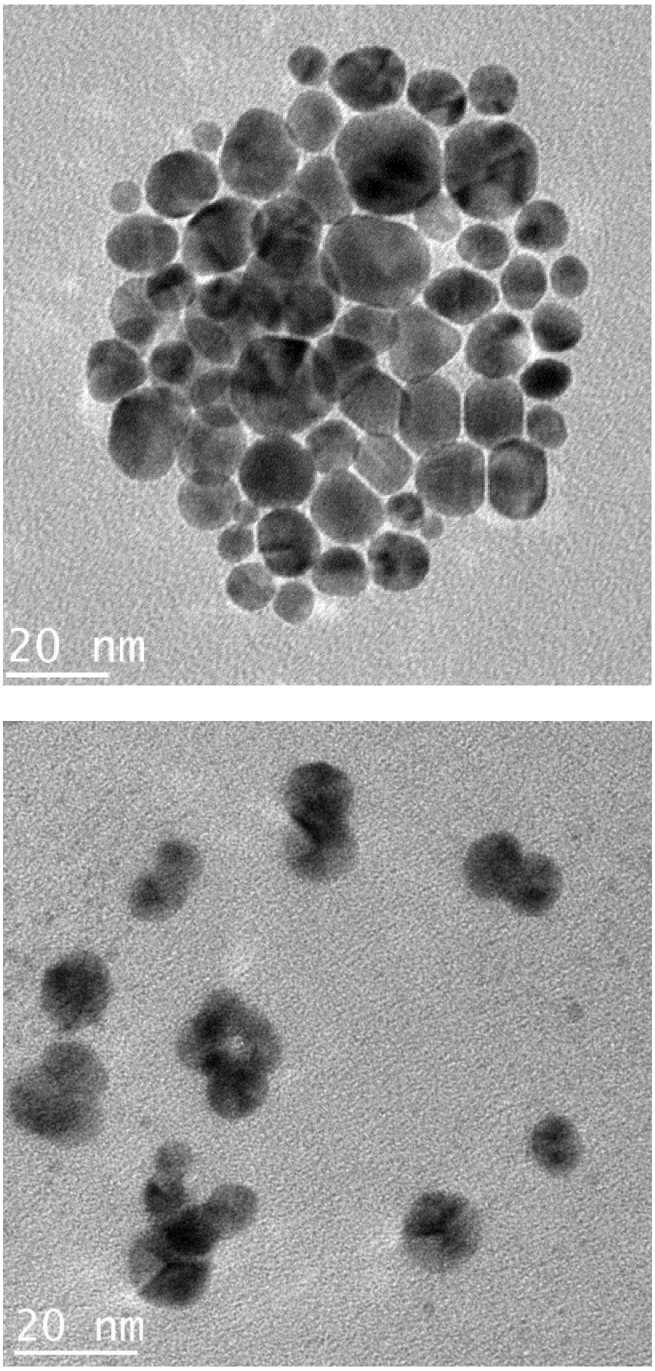

Fig. S 41. TEM images of **4d**.

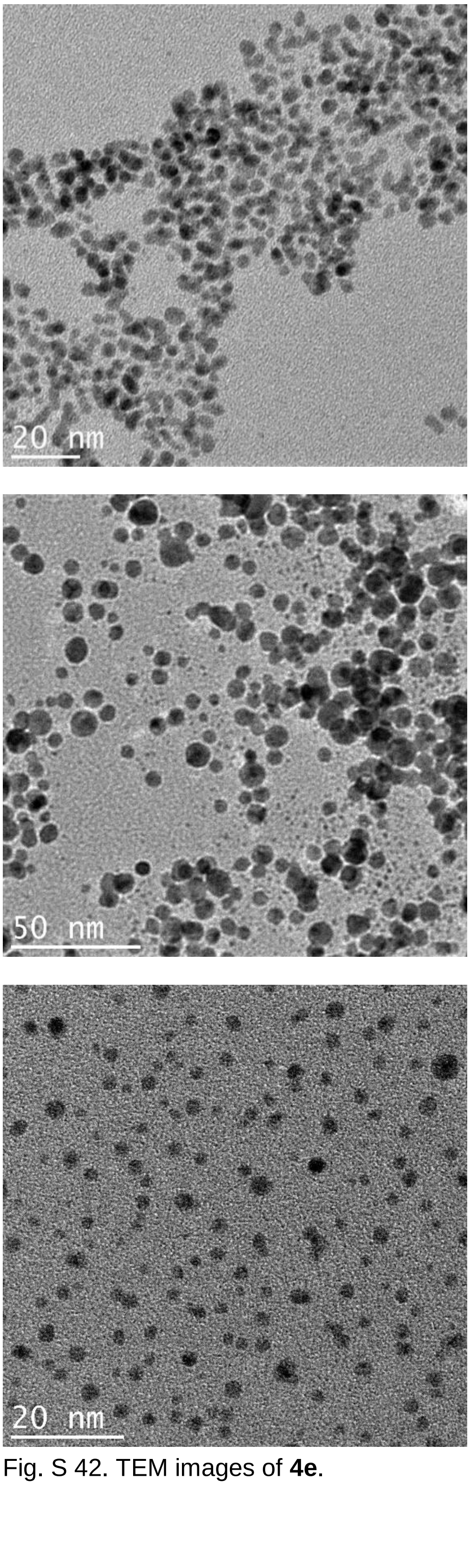

Fig. S 42. TEM images of **4e**.

References

[1] R. Uson, A. Laguna, M. Laguna, D. A. Briggs, H. H. Murray, J. P. Fackler, John Wiley & Sons, Ltd, **2007**, pp. 86.

[2] A. S. K. Hashmi, C. Lothschütz, C. Böhling, T. Hengst, C. Hubbert and F. Rominger, *Advanced Synthesis & Catalysis*, 2010, **352**, 3001-3012.

[3] A. J. Young, C. J. Serpell, J. M. Chin, M. R. Reithofer, *Chem. Commun.* **2017**, *53*, 12426–12429.
